# Machine Learning-Based Virtual Screening of Antibacterial Agents against Methicillin-Susceptible and Resistant Staphylococcus aureus

**DOI:** 10.1021/acs.jcim.4c00087

**Published:** 2024-03-04

**Authors:** Philipe Oliveira Fernandes, Anna Letícia Teotonio Dias, Valtair Severino dos Santos Júnior, Mateus Sá Magalhães Serafim, Yamara Viana Sousa, Gustavo Claro Monteiro, Isabel Duarte Coutinho, Marilia Valli, Marina Mol Sena Andrade Verzola, Flaviano Melo Ottoni, Rodrigo Maia de Pádua, Fernando Bombarda Oda, André Gonzaga dos Santos, Adriano Defini Andricopulo, Vanderlan da Silva Bolzani, Bruno Eduardo Fernandes Mota, Ricardo José Alves, Renata Barbosa de Oliveira, Thales Kronenberger, Vinícius Gonçalves Maltarollo

**Affiliations:** † Departamento de Produtos Farmacêuticos, Faculdade de Farmácia, 260139 Universidade Federal de Minas Gerais (UFMG), Belo Horizonte, Minas Gerais 31.270-901, Brazil; ‡ Departamento de Microbiologia, Instituto de Ciências Biológicas, Universidade Federal de Minas Gerais (UFMG), Belo Horizonte, Minas Gerais 31.270-901, Brazil; § Departamento de Química Orgânica, Instituto de Química, 153998 Universidade Estadual Paulista (UNESP), Araraquara, São Paulo 14.800-900, Brazil; ∥ Departamento de Física e Ciência Interdisciplinar, Instituto de Física, Universidade de São Paulo (USP), São Carlos, São Paulo 13.563-120, Brazil; ⊥ Departamento de Fármacos e Medicamentos, Faculdade de Ciências Farmacêuticas, Universidade Estadual Paulista (UNESP), Araraquara 14.800-903, Brazil; # Departamento de Análises Clínicas e Toxicológicas, Faculdade de Farmácia, Universidade Federal de Minas Gerais (UFMG), Belo Horizonte, Minas Gerais 31.270-901, Brazil; ∇ Institute of Pharmacy, Pharmaceutical/Medicinal Chemistry and Tübingen Center for Academic Drug Discovery, 9188 Eberhard Karls University Tübingen, 72076 Tübingen, Germany; ○ School of Pharmacy, Faculty of Health Sciences, University of Eastern Finland, 70211 Kuopio, Finland

## Abstract

The application of computer-aided drug discovery (CADD) approaches has enabled the discovery of new antimicrobial therapeutic agents in the past. The high prevalence of methicillin-resistantStaphylococcus aureus­(MRSA) strains promoted this pathogen to a high-priority pathogen for drug development. In this sense, modern CADD techniques can be valuable tools for the search for new antimicrobial agents. We employed a combination of a series of machine learning (ML) techniques to select and evaluate potential compounds with antibacterial activity against methicillin-susceptible S. aureus (MSSA) and MRSA strains. In the present study, we describe the antibacterial activity of six compounds against MSSA and MRSA reference (American Type Culture Collection (ATCC)) strains as well as two clinical strains of MRSA. These compounds showed minimal inhibitory concentrations (MIC) in the range from 12.5 to 200 μM against the different bacterial strains evaluated. Our results constitute relevant proven ML-workflow models to distinctively screen for novel MRSA antibiotics.

## Graphical abstract

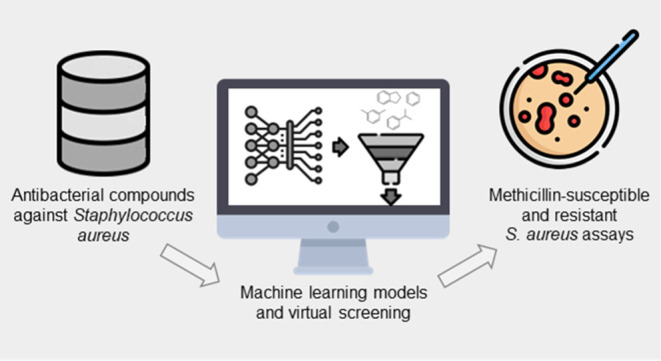

## Introduction

Infections with antimicrobial-resistant (AMR) and multidrug-resistant (MDR) bacteria are a persistent and critical problem to public health worldwide. The report of a virtually untreatable case of Escherichia coli infection in the United States back in 2014 showed the current emergence of MDR bacteria, whereas there is a lack of newly approved antibiotics to contain such drug-resistant infections.^1^ Only in the US, at least 2.8 million people become infected with bacteria that harbor some type of resistance to commercially available antibiotics, of whom 35,000 die each year.

Among the most concerning MDR bacteria are strains of methicillin-resistant Staphylococcus aureus (MRSA) that have an alternative form of the penicillin-binding protein (PBP), named PBP2a, with lower affinity for all β-lactams (penicillins, cephalosporins, and carbapenems), and β-lactamases producing Enterobacteriaceae, such as Escherichia coli expressing TEM-1 β-lactamase, which hydrolyzes penicillins and early cephalosporins.^2,3^ Ultimately, it is estimated an increase of US$ 1.7 billion in healthcare costs annually only to treat MRSA-infected patients and another US$ 1.2 billion to manage infections with β-lactamase producing Enterobacteriaceae.^4^ In 2019, AMR has been listed among the 10 global threats by the World Health Organization (WHO).^5^ In the US, mortality by MRSA infection already surpasses the number of deaths from HIV/AIDS and tuberculosis combined,^6^ escalating this threat by the Centers of Disease Control and Prevention (CDC)^4^ and WHO^7^ as a high-priority pathogen for the search and development of new drugs.

Taking these into consideration, the discovery of novel antibiotic classes, low toxicity, and less susceptibility to resistance mechanisms is increasingly urgent.^8,9^ Nevertheless, the main challenges in the development of new antibacterial drugs include high costs and low approval rates by health agencies,^10^ which demand reliable strategies for hit identification. In this sense, approaches employing machine learning (ML) techniques can help in the identification of new antibacterial hits. ML algorithms can solve nonlinear problems and predict multiple end points, such as pharmacokinetic and toxicity properties combined in a single model.^11–14^ Also, the combination of distinct models can improve the robustness and reliability.^15–17^ For example, halicin is a structurally novel class of antibiotic discovered using a combination of deep learning models and *in vitro* validation.^18^ Halicin presented potent broad-spectrum antibacterial activity both *in vitro* and in murine models against both standard and clinically isolated strains. Further applications of this convolutional model graph to virtually screen the ZINC15 ^19^ database, containing >107 million entries, resulted in eight new hits with bacteriostatic effect over at least one Gram-positive (S. aureus) or Gram-negative (E. coli) bacteria.

Despite its relevance, few models focused on identifying new hits against MRSA.^20^ In this regard, we generated multiple machine learning-based models to predict compounds with potential antibacterial activity. Those models were generated using diverse methods such as distance-based, neural networks, and support vector machines and were validated according to QSAR literature standards. Validated models were used to virtually screen two relevant small molecule libraries BraCoLi^21^ and NuBBE.^22,23^ Potential hits selected from this screening underwent biological evaluation against an S. aureus reference strain as well as a reference and two clinical isolates of MRSA strains. We provide here an original ML classification-based workflow to screen novel hits against MRSA and MSSA.

## Methods

### Data Curation

The compounds with antibacterial activity were retrieved from the ChEMBL database^24^ version 24.1 (accessed on April 24th, 2018) using the target ID CHEMBL352 (“S. aureus”), resulting in 134,510 entries. Sixty-six compounds from the literature were also added to the data set.^25,26^ All strains present in the database were manually curated, and the compounds were classified as methicillin-resistant S. aureus (MRSA) or methicillin-susceptible S. aureus (MSSA), based on the libraries of American Type Culture Collection (ATCC), The Bacterial Diversity MetaDabase (BacDive), China Center of Industrial Culture Collection (CICC), Microbial Type Culture Collection (MTCC), and German Collection of Microorganisms and Cell Cultures (DSMZ), as well as on scientific articles and other publications such as the Food and Drug Administration (FDA) summary.

For our ML models, we considered only precise activity values; *i.e.*, entries with inconvertible units such as inhibition percentage and millimeters in the inhibition halo were discarded. The data set was prepared using the RDKit^27^ nodes implemented in the KNIME Analytics Platform^28^ using the following process: (i) all precise activity values were converted into μM, and a cutoff of 50 μM was defined to arbitrarily classify compounds as active (<50 μM) or inactive (>50 μM); (ii) compounds with more than one entry present in both active and inactive groups were removed from the data set, alternatively for duplicated in the same class only one entry was kept; (iii) unannotated racemic mixtures and salts were removed, and only organic compounds were maintained by atomic-type filtering (H, C, O, F, Br, I, Cl, P, and S). This process was made three times, resulting in three subsets: MSSA inhibitors (12,331 unique compounds), MRSA inhibitors (3195 unique compounds), and a consensus subset (2009 unique compounds) comprising compounds with active and inactive labels for both MSSA and MRSA in a convergent way (named as sensible-resistant intersection (SRI)).

### Data Set Preparation

These structures were submitted to the calculation of unidimensional and bidimensional descriptors as well as to molecular fingerprints (Klekota Roth, MACCS, PubChem, Atom Pairs 2D, Substructure, and EState) using the PaDEL Descriptor software.^29^ Prior to the descriptor calculations, the data sets had their conformers generated using OMEGA 2.5.1.4 ^30,31^ and had their ionization state corrected to pH 7.4 using the *fixpka* option in the QUACPAC 1.6.3.1 software.^32^

### Training and Test Split

Each data set was rationally divided into training and test sets in an 80:20 ratio using a preliminary version of the MASSA algorithm.^33^ Briefly, this is based on Hierarchical Cluster Analysis (HCA) of three spaces: (i) drug-like properties (molecular weight, calculated *n*-octanol/water partition coefficient, number of hydrogen bond acceptors and donors, number of rotatable bonds, sp^3^ carbon ratio, and topological polar surface area); (ii) structural diversity, measured by PubChem fingerprints; and (iii) biological activity.

### Feature Selection

The selection of relevant descriptors prior to training the models was divided into two steps: variable exclusion and selection following a previously published strategy.^34^ In the exclusion stage, all features containing missing values and linearly correlated variables were excluded. For the selection stage, three strategies were used:

#### Fisher Weight (FW)

The FW estimates the relative importance of each feature using the distance of a variable between classes, which is given by the difference of the average of the values of the variable in each class, divided by the sum of the variances of the variable in each class, and can be interpreted as the normalized distance between classes^35,36^ (eq 1).

1 $$\mathrm{FW}=\frac{{\left[X_{i}\left(1\right)-X_{i}\left(2\right)\right]}^{2}}{\left[S_{i}^{2}\left(1\right)+S_{i}^{2}\left(2\right)\right]}$$

#### Random Forest (RF)

It is a ML algorithm based on the construction of multiple decision trees. A default run was performed, and the features were ranked according to their contributions. Two strategies were applied using only the contribution at level 0 (RF selection) and the weighted contribution of levels 0 and 1 (wRF selection).

#### Principal Component Analysis (PCA)

This technique is an orthogonal linear transformation into a new coordinate system, retaining the maximum variance from the original variables.^37^ For feature selection, two principal components (PC) were used and their scores were considered to rank the variables. Two strategies were applied using only the first component (PCA selection) and the weighted contribution of the first and second components (wPCA selection).

Each strategy leads to a five-ranked variable list (FW, PCA, wPCA, RF, and wRF), which was used to construct the inputs for the machine learning models. The inputs were composed of 5, 10, 15, or 20 well-ranked variables from each selection process.

### Machine Learning (ML) Model Selection and Generation

The classification ML models were built and validated also using the KNIME Analytics Platform. We performed internal and external validations as well 5-fold cross-validation, using Matthews Correlation Coefficient (MCC, eq 2, ranging from −1 to 1), F1-Score (eq 3), the area under the curve (AUC) of a receiver operating characteristic curve (ROC), true positive rate (TPR), and true negative rate (TNR) metrics to evaluate the generated models.

2 $$\mathrm{MCC}=\frac{\mathrm{TP}\times \mathrm{TN}-\mathrm{FP}\times \mathrm{FN}}{\sqrt{\left(\mathrm{TP}+\mathrm{FP}\right)\left(\mathrm{TP}+\mathrm{FN}\right)\left(\mathrm{TN}+\mathrm{FP}\right)\left(\mathrm{TN}+\mathrm{FN}\right)}}$$

3 $$F1‐s\mathrm{core}=\frac{2\mathrm{TP}}{2\mathrm{TP}-\mathrm{FP}-\mathrm{FN}}$$

where TP = true positives; TN = true negatives; FP = false positives; FN = false negatives.

The resulting models were ranked primarily according to the MCC, followed by F1-Score and AUC. Each ML algorithm (listed in the Table 1) was heuristically evaluated by varying an intrinsic set of hyperparameters for the model optimization.^38^ Their applicability domain was calculated by measuring the distance between the training and test sets following a bounding box approach previously described^39^ using Scikit-learn^40^ and SciPy^41^ libraries. The training and test data were separated and clustered by PCA. A sample was considered inside the domain if its distance from the training data was less than the threshold of 95%. The same procedure was also applied to hits from the virtual screening, where they were compared to the training set.

**Table 1:** Description of Each Machine Learning Algorithm and the Parameters Employed in Virtual Screening

machine learning algorithm	hyperparameters
k-nearest neighbors (kNN)	the number of nearest neighbors (k) from 1 to 27 in odd numbers.
multilayer perceptron (MLP)	the number of layers, from 1 to 5 in steps of 1 layer and the number of hidden neurons per layer, from 20 to 100 in steps of 20 neurons.
naïve Bayes (NB)	the probability from 0.1 to 5 in 0.1 steps during the NB model optimization.
decision tree (DT)	the minimum number of records per node is from 2 to 100.
random forest (RF)	the number of levels from 10 to 50 in steps of 10 levels, and the number of models from 10,000 to 50,000 in 10,000 steps.
support vector machine (SVM)	polynomial: bias 1 to 5 in 1 step, and γ 1 to 5 in 1 step.
	RBF: sigma from 0.5 to 5 in 0.5 steps, from 10 to 50 in 5 steps, and from 100 to 300 in 50 steps.

### Virtual Screening

After the training and validation step, the three most predictive models for the MRSA inhibitor subset, the MSSA inhibitor subset, and the SRI subset were selected for a virtual screening protocol, totaling nine distinct virtual screenings. Then, those models were employed to predict the activities of BraCoLi^21^ and NuBBE^22,23^ databases. After the compounds were classified as active by most of the models, a concordance between each subset was made, and the selected compounds were experimentally validated according to the availability of samples.

### *In Vitro* Assays

E. coli (ATCC n° 35128) – a TEM-1 β-lactamase producing strain,^42^ S. aureus (ATCC n° 29213) – herein referred to as MSSA, MRSA (ATCC n° 43300), and two clinical strains of MRSA (n° 5749 and 6154 ^43^) were used in the biological assays. The antibacterial activity and minimal inhibitory concentration (MIC) of the screened compounds were evaluated with the broth microdilution method in 96-well microplates, according to the Clinical and Laboratory Standards Institute (CLSI) protocol.^44^ Initially, compounds were evaluated in a screening assay to identify promising hits. The compounds were dissolved in dimethyl sulfoxide (DMSO) and then diluted in Mueller Hinton broth (MHB; Oxoid, Thermo Scientific, U.K.) to a concentration of 200 μM. After the addition of 100 μL to each well, the same volume of bacterial suspension containing 10^5^ CFU/mL was added to each of the previous solutions, resulting in a final compound concentration of 100 μM. A viability control (bacterial suspension only), inhibition controls (MHB containing five times the MIC of penicillin G for MSSA, vancomycin for MRSA strains, and streptomycin for E. coli), a sterility control (medium only), and a vehicle control (DMSO 2% v/v) were included in each assay microplate. After 24 h of incubation at 37 °C, the microplates were visually inspected for inhibition of bacterial growth, and the absorbance at 600 nm of each well was read using a microplate reader spectrophotometer (VersaMax, Molecular Devices, CA). The percentage of growth inhibition was calculated as the percentage of growth reduction compared with the vehicle control. Wells showing no visual bacterial growth (no turbidity) were considered hits. The MIC of the promising compounds in the initial screening were tested as previously described, with a serial dilution of each compound (100–1.56 μM). All conditions were tested in triplicate from at least three independent assays.

As for the cytotoxic concentration of 50% (CC_50_) determination, the Vero (ATCC CCL-81) cell line was cultured in Eagle’s Minimum Essential Medium (MEM) (Cultilab, Brazil). The medium was supplemented with 5% fetal bovine serum (FBS) (Cultilab, Brazil), in addition to 100 IU/mL penicillin (Cellofarm, Brazil), 100 μg/mL streptomycin (Merck, Germany), and 0.25 μg/mL amphotericin B (Cultilab, Brazil). Cells were seeded in 96-well microplates (4.0 × 10^4^ cells per well) and incubated at 37 °C and 5% CO_2_ atmosphere. After 24 h of incubation, 200 μL of fresh medium containing a serial dilution of the compounds (100–1.56 μM) was added to the plates. Serial dilutions of DMSO were used as vehicle controls. After 72 h of incubation under the same conditions, 100 μL of 3-(4,5-dimethylthiazol-2-yl)–2,5-diphenyltetrazolium bromide (MTT)^43^ in MEM (0.5 mg/mL) was added to each well. After 3 h of incubation with MTT (ThermoFischer Scientific), the medium was removed, and 100 μL of DMSO was added to each well to solubilize the formazan crystals. After a brief incubation under shaking for 20 min, the absorbance at 570 nm of each well was read using a spectrophotometer (VersaMax, Molecular Devices). The percentages of inhibition of cell viability were calculated by using the vehicle control. The cytotoxic concentration of 50% (CC_50_) is defined as the lowest concentration of a given compound that can reduce the viability of cultured cells by 50%. Posteriorly, the selectivity index (SI), that is, the ratio between the CC_50_ and MIC values of each active compound, was calculated. All conditions were tested in triplicate and at least two independent assays. The overall procedures applied in this work are illustrated in Figure 1.

**Figure 1:**
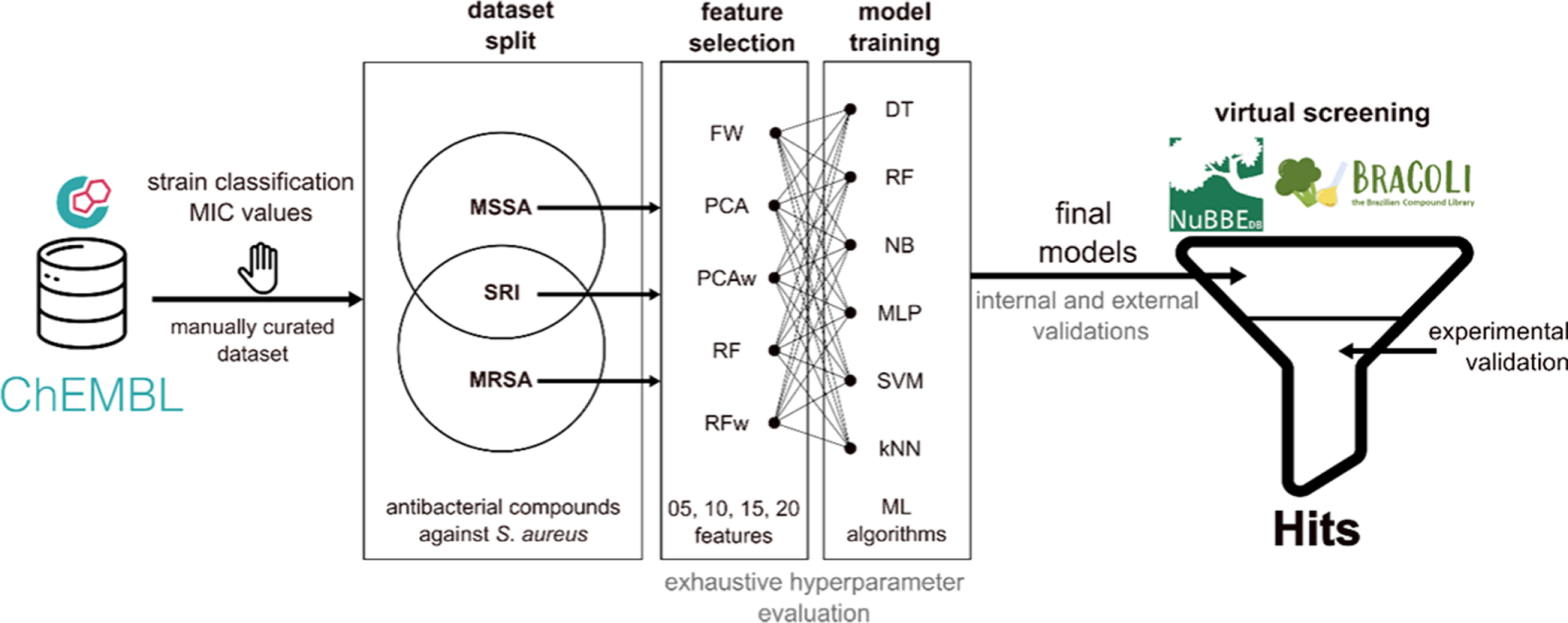
Illustration of the overall workflow applied in this work. After data curation from the ChEMBL database, the strains were classified as MRSA, MSSA, and SRI. Features calculated using PaDEL descriptors were selected with three different methods prior to the ML algorithm training. Six techniques were employed to generate models for the subsets of each end point (MRSA, MSSA, and SRI). After that, two virtual screenings were performed on BraCoLi and NuBBE data sets, following *in vitro* assays against MSSA, MRSA, and E. coli strains.

## Results and Discussion

### Data Curation

The curation process began with the categorization of the S. aureus strains in the ChEMBL antibacterial data as either MRSA or MSSA. Among all entries, almost 44% had no strain declared and, in comparison, the most represented strain was S. aureus ATCC 25923 with only 7% of the total entries (Supporting Figure S1). Altogether, 74 strains were classified as MRSA and 85 as MSSA. The manually curated list of MRSA and MSSA strains is provided in Supporting Table S1.

### Machine Learning Models

Prior to training our models with ML algorithms, feature selection was employed to reduce noise and prevent overfitting in the models.^45^ QSAR models usually had a limited number of samples and features; however, the use of computational descriptors drastically increased the available numbers, increasing the complexity of the process of choosing the relevant variables to model the problem. Also, from the QSAR principles defined by the OECD, a QSAR model should have a number of variables/descriptors that allow its interpretability.^46^ In that sense, the following strategies for feature selection were employed: Fisher’s weight, PCA, weighted PCA, RF, and weighted RF. This resulted in five distinct feature lists for each end point (MRSA, MSSA, and SRI) with low similarity between them (Supporting Figure S2).

Initially, ML algorithms and their hyperparameters were employed to generate the models for the 20 subsets for each end point (MRSA, MSSA, and SRI). Then, the first step used to find the most robust and predictive model was to analyze the average performance produced by each algorithm. Thenceforward, the average performance among the feature selection methods was analyzed, followed by an analysis of the number of features used to build the models. Finally, the performance of the generated models was observed by intrinsic hyperparameter screening to identify the most robust and predictive model.

Each ML algorithm and its intrinsic hyperparameters were optimized according to their MCC values. The kNN algorithm outperformed other methods for the MSSA inhibitor subset (Figure 2A) and the kNNw algorithm outperformed other methods for MRSA inhibitors and SRI subsets (Figure 2B,C). As our data set is structurally diverse, a distance-based method would be the most recommended to classify compounds as active and/or inactive antibacterial.

**Figure 2:**
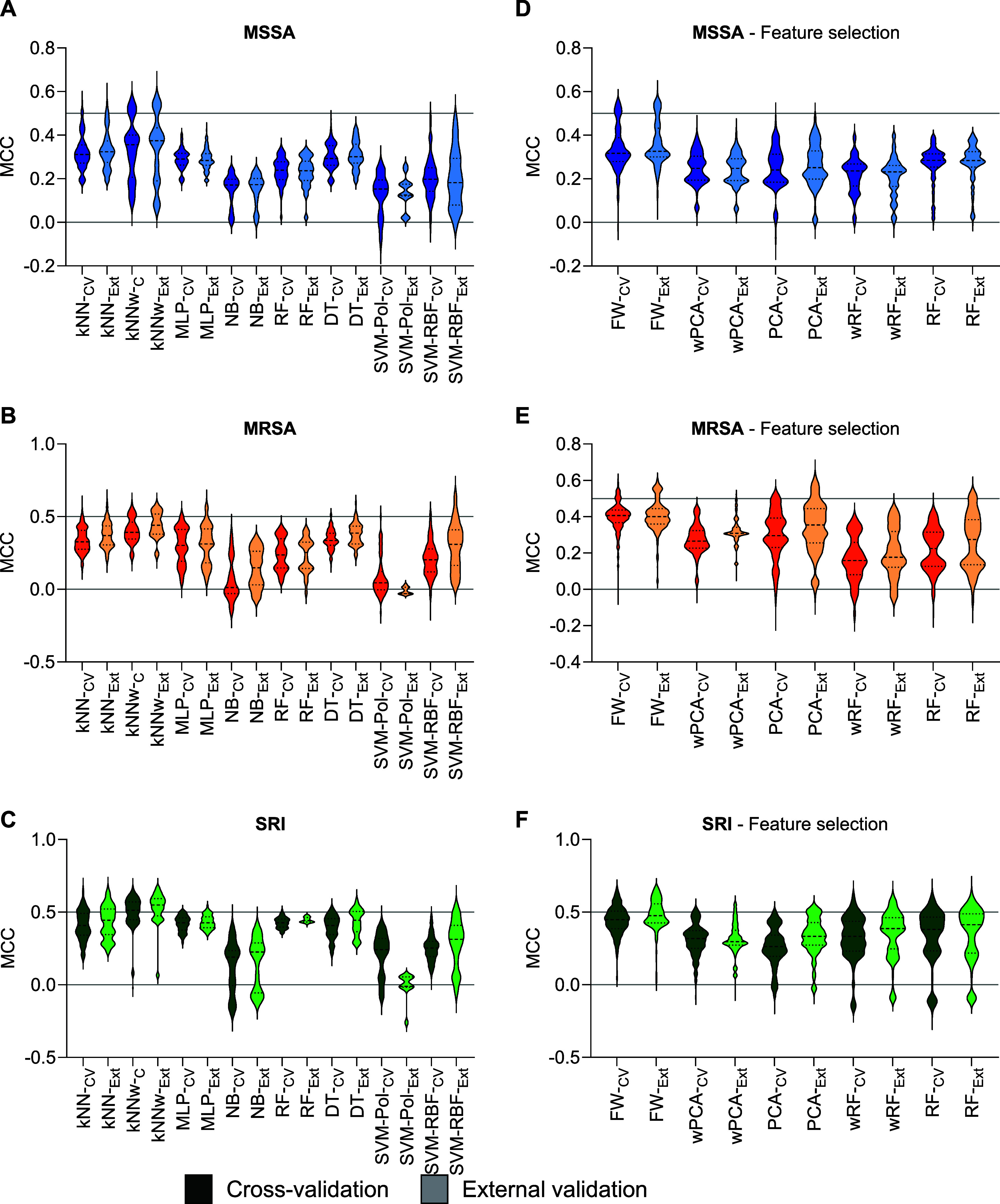
Average of the Matthews Coefficient Correlation (MCC) in the 5-fold cross-validation (_CV_MCC) and external validation (_EXT_MCC) for different models: machine learning algorithms; k-nearest neighbors (kNN), weighted k-nearest neighbors (wkNN), multilayer perceptron (MLP), naïve Bayes (NB), decision trees (DT), and random forest (RF) (A–C). Feature selection methods; Fisher weight (FW), principal component analysis (PCA), weighted principal component analysis (wPCA), random forest (RF), and weighted random forest (wRF) (D–F). The results were also separated by subsets: MSSA (A,D, blue), MRSA (B,E, orange), and SRI (C,F, green). Data from cross-validation are depicted in dark colors, while external validation results are displayed in lighter shades.

In combination with the generation of models by heuristically searching the intrinsic hyperparameters of each method, the performance of each feature selection strategy was evaluated (Figure 2D–F). Interestingly, the employment of variables selected by Fisher Weight produced more robust and predictive models than other methods for all subsets. This result corroborates previous QSAR studies using several variable selection methods.^34^ In addition, the maximum tested number of variables (20 descriptors) produced the most robust and predictive models (Figure 3A–C).

**Figure 3:**
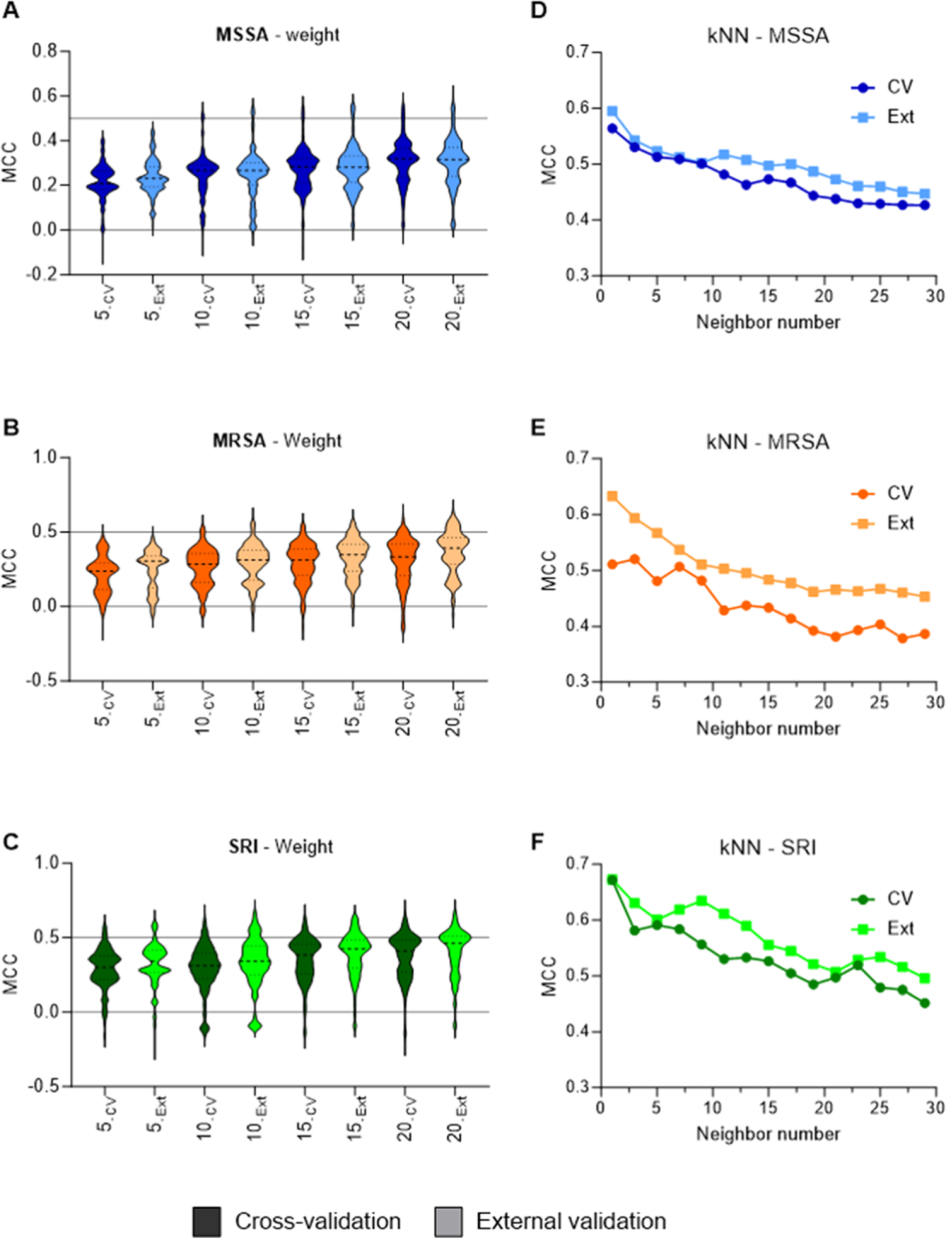
(A–C) Number of features per model. (D–F) Number of neighbors in the kNN model. Data sets are splitted by MSSA (A,D, blue), MRSA (B,E, orange), and SRI (C,F, green). Data from cross-validation are depicted in dark colors, while external validation results are displayed in lighter shades.

Finally, to access the most robust and predictive model, the influence of hyperparameters on internal and external validations was evaluated. Regarding the kNN methods, the only evaluated hyperparameter is the number of neighbors. For the three subsets, a decrease in the MCC values was observed as the number of neighbors increased (Figure 3D–F). This suggests that the active compounds should be arranged in smaller groups. Since the data sets are structurally diverse, the expansion in the chemical space can lead to the incorporation of inactive compounds, decreasing the robustness of the model. One hypothesis for that observed trend in addition to the chemical diversity is the presence of compounds with distinct antibacterial mechanisms of action in the data sets.^47^ For that reason, models containing only one neighbor were selected. For the virtual screening campaign, in addition to the kNN models, the second and third robust test models for each end point were also selected intending to perform a consensus prediction (Table 2). This selection of an optimum model maximizing both internal and external MCC values was done for other machine-learning-method-derived models (in this specific case, DT, MLP, and SVM, Table 2) but, due to the higher dimensionality of hyperparameters, the step-by-step analyses were not shown.

**Table 2:** Method, Cross-Validation MCC (_CV_MCC), External Validation MCC (_EXT_MCC), Cross-Validation AUC (_CV_AUC), External Validation AUC (_EXT_AUC), Cross-Validation F1-Score (_CV_F1), and External Validation F1-Score (_CV_F1) for the Selected Models for Each Subset

subset	method	_CV_MCC	_EXT_MCC	_CV_AUC	_EXT_AUC	_CV_F1	_EXT_F1
MSSA	kNN	0.564	0.595	0.782	0.797	0.773	0.790
	kNNw	0.557	0.595	0.779	0.797	0.768	0.790
	DT	0.475	0.445	0.788	0.794	0.721	0.708
MRSA	kNN	0.607	0.633	0.843	0.867	0.835	0.860
	MLP	0.523	0.613	0.818	0.863	0.805	0.884
	SVM	0.571	0.644	0.832	0.830	0.822	0.860
SRI	kNNw	0.607	0.617	0.802	0.806	0.862	0.859
	kNN	0.672	0.673	0.832	0.836	0.882	0.878
	DT	0.492	0.598	0.807	0.846	0.830	0.850

The visualization of chemical space using the descriptors of the best model for each subset clearly indicates the nonlinearity of all systems (Supporting Figure S3). In this sense, this observation corroborates the employment of ML methods to model the selected subsets rather than linear traditional QSAR techniques. Furthermore, the nonlinearity of data sets also suggests the difficulty of establishing a general structure–activity relationship, and it is expected due to the biological diversity of samples present in each subset comprising compounds with potential distinct mechanisms of action. However, the feature distribution between classes of the SRI model was analyzed in order to establish a model interpretation. The distributions were very similar and there was an overlapping of descriptors for active and inactive compounds (Supporting Figure S4). However, the frequency of ExtFP350, PubchemFP390, PubchemFP545, and PubchemFP656 was higher in the active compounds (Figure 4) suggesting the importance of those features to antibacterial activity. The PubChem fingerprints bits are respectively C­(:N)­(:N), O–C:C–O, and O–C–C-C:C where the colon represents an aromatic bond, and it could be related to physicochemical properties. Despite the knowledge that the ExtFP350 is higher in the active compounds, due to the software implementation and documentation, it is not possible to determine this feature. The full descriptor list for the SRI data set as well MRSA and MSSA is described in Supporting Table S2.

**Figure 4:**
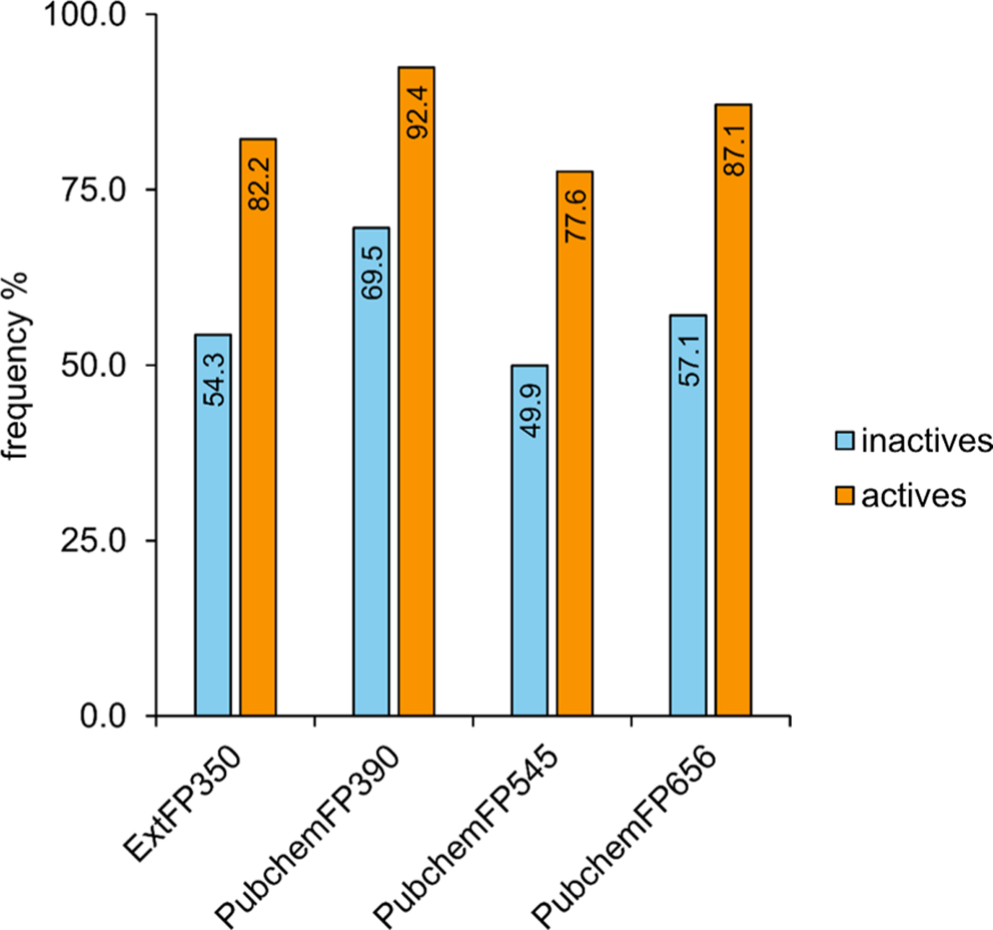
Feature distribution among active and inactive compounds from the SRI data set.

### Virtual Screening

After validation of the ML models, all molecules in the BraCoLi and NuBBE databases had the descriptors calculated and were submitted to the prediction of the models described in Table 2. The compounds were considered as a positive prediction when at least two models per end point predicted it as active. Also, available compounds classified as inactive were tested as negative control. In total, sixty-two compounds were submitted to the experimental assays classified as follows: 22 positive/40 negative from MSSA models; 29 positive/33 negative from MRSA models; and 30 positive/32 negative from the SRI models. In addition, more than 98% of all compounds submitted to experimental validation were classified inside the applicability domain in concordance with training/test sets applicability domain distribution (Supporting Table S3 and Figure S5). Table 3 displays the hits from the virtual screening, and the remaining experimentally tested compounds are described in Supporting Table S4.

**Table 3:**
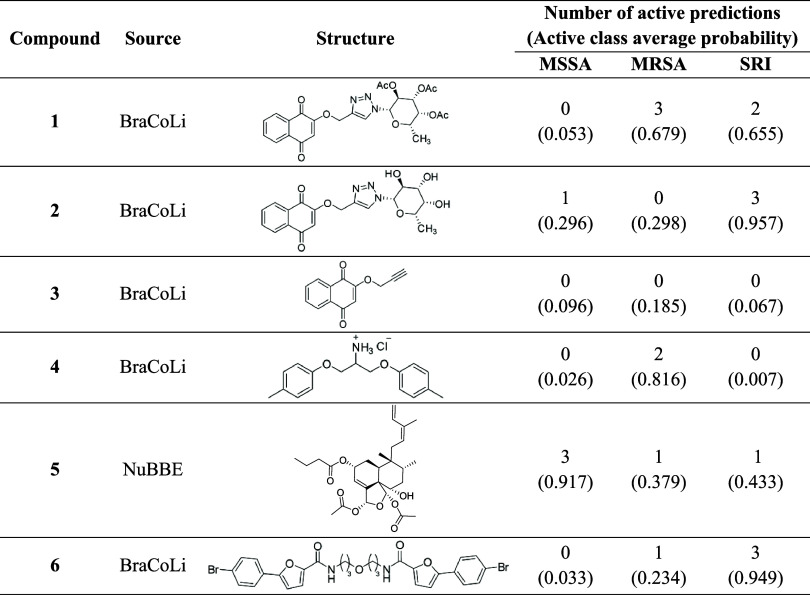
Hits in Virtual Screening Selected for the *In Vitro* Assays

The literature shows that the use of combined predictions can improve the predictability of QSAR models.^15,16^ Following this strategy, we successfully found active compounds against bacteria. Also in our case, attention to the probabilities of the predictions was relevant. As an example, compound **1** was predicted as inactive against MSSA, but the probability for the three models was less than 16%, indicating low confidence in the predictions of these models. Also, a similar situation happened to compound **2** where the two inactive MSSA predictions had probabilities equal to zero. So, in similar cases, the probabilities of the predictions may be used together to prioritize the compound selection.

### Identified Hit Compounds Inhibit MRSA and MSSA Growth *In Vitro*

Among the 62 compounds initially tested, six of them (described in Table 4) presented bacterial growth inhibition >90.0% at 100 μM in the initial screening against MSSA and MRSA reference strains, in addition to only one compound (**4**) showing some activity against E. coli (85.5% inhibition). The synthesis/isolation procedures as well as the proper chemical characterization of compounds **1** (2-[1-(2,3,4-tri-*O*-acetyl-6-deoxy-β-l-galactopyranosyl)-1,2,3-triazol-4-(methyl)­oxy]-1,4-naphtoquinone),^48^ **2** (2-[1-(6-deoxy-β-l-galactopyranosyl)-1,2,3-triazol-4-(methyl)­oxy]-3-(3-methyl-2-butenyl)-1,4-naphtoquinone),^48^ **3** (2-[(prop-2-yn-1-yl)­oxy]-1,4- naphtoquinone),^48^ **4** (1,3-bis­(4-methylphenoxy)­propano-2-aminium chloride),^49^ and **5** (caseargrewiin F, [(1*S*,­3*R*,­5*R*,­6a*S*,­7*R*,­8*R*,­10*S*,­10a*S*)-1,3-diacetyl­oxy-10-hydroxy-7,8-dimethyl-7-[(2*Z*)-3-methyl­penta-2,4-dienyl]-1,­3,­5,­6,­6a,­8,­9,­10-octahydro­benzo­[*d*]­[2]­benzofuran-5-yl] butanoate)^50^ were already reported in the literature. Although compound **4** characterization is reported in the literature, we provided the NMR spectra due to the absence of this data in the original paper for comparison purposes. In addition, compound **6** (*N*,*N*′-(oxybis­(propane-3,1-diyl))-bis­(5-(4-bromophenyl)­furan-2-carboxamide)) is novel and the spectroscopic data is described in the characterization data section of the Supporting Information as well as the synthetic procedure. Compounds **3, 4**, and **6** have qualitative purity determined higher than 95% due to the absence of impurity signals on the reported spectra. Also, compounds **1** and **2** have purities equal to 98 and 97%, respectively, determined by UPLC,^48^ and compound **5** has purity of 98.6% determined by HPLC.^50^

**Table 4:** Minimum Inhibitory Concentration (MIC), Percentage of Inhibition, Cytotoxic Concentration of 50% (CC_50_), and Selectivity Indexes (SI) of the Compounds Selected by the ML Models against MSSA, MRSA Strains, and E. coli

		compounds					
*in vitro* assays		**1**	**2**	**3**	**4**	**5**	**6**
MIC (μM) (% of growth inhibition)	MSSA (ATCC 29123)	**100** (99.60)	**12.5** (96.08)	**50** (96.52)	**50** (97.80)	**200** (96.43)^a^	**50** (>99.9%)
						**100** (27.39)	
	MRSA (ATCC 43300)	**>100** (70.02)	−	**50** (>99.9)	**100** (90.38)	**100** (>99.9)	**100** (>99.9)
							**50** (85.33)
	MRSA (5749)	**>100** (41.76)	−	**50** (>99.9)	−	−	**100** (>99.9)
							**50** (73.47)
	MRSA (6154)	**>100** (50.60)	−	**50** (>99.9)	−	**100** (>99.9)	**100** (>99.9)
							**50** (78.41)
	E. coli (ATCC 35218)	−	−	−	**100** (87.0)	−	−
CC_50_ (μM)	Vero cells	33.75 ± 3.44	>200	21.16 ± 8.89	19.03 ± 6.10	ND	43.44 ± 4.66
SI	MSSA (ATCC 29123)	0.33	>16	0.42	0.38	ND	0.87
	MRSA (ATCC 43300)	<0.33	−	0.42	0.19	ND	0.43
	MRSA (5749)	<0.33	−	0.42	−	ND	0.43
	MRSA (6154)	<0.33	−	0.42	−	ND	0.43
	E. coli (ATCC 35218)	−	−	−	0.19	ND	−

a **5** was submitted to 200 μM in order to evaluate it in comparison to the MRSA results; - no antibacterial growth in the tested concentrations.

After the initial screening, these hit compounds were submitted to MIC determination using a broth microdilution assay (Table 4). Here, **2** showed the lowest MIC values (12.5 μM), but only against MSSA. In addition, **3**, **6**, and **1** showed MIC values (50 μM; 50 to 100 μM; and 100 to >100 μM, respectively) against MSSA and all MRSA strains. Curiously, compound **5** was the only compound showing higher MIC values for MSSA (200 μM) in comparison to MRSA ATCC and 6154 strains (100 μM). Finally, **4** was the only compound with MIC values against E. coli (100 μM), while also being active against MSSA (50 μM) and MRSA (100 μM) reference strains. The remaining tested compounds are described in Supporting Table S4.

Interestingly, **3** is a smaller analogue of **1** and **2**, without acetoxyl or hydroxyl substituents. It retains antibacterial activity, such as 2-fold lower MIC values against MRSA strains in comparison to **1**. Comparison between active compounds **1**, **2**, and **3** and inactive tested compounds reveals that 1,4 naphthoquinone is key for activity, whereas lawsone/lapachol moieties (present in compounds S22–S27, S30, S31, and S34–S36, Supporting Table S4) lead to inactive compounds. Additionally, the triazole linker, in bivalent quinone-sugar compounds, seems to be key, as directly liked quinone and sugars were inactive (compounds S28 and S29, Supporting Table S4). We attribute the activity to the sugar moiety of those compounds as the triazole linker alone has no antibacterial activity (compound S12, Supporting Information Table S4). This activity is very sensible to the type of sugar; it can be seen in the potency difference between the hits and in the other inactive compounds (compounds S1, S4, S7, S8, S21–S27, and S33, Supporting Table S4)

On the other hand, the higher lipophilicity of **4** among the six active compounds could support it being the only one active against Gram-negative bacteria, since it would be able to permeate cellular membranes more easily.^51,52^ Considering compound **6**, another symmetric compound such as **4**, one could argue that a longer spacer (*i.e.*, more carbon atoms) could still be important for antibacterial activity^53^ and thus corroborate the activity of this compound being slightly broader to MRSA strains and consistent against MSSA and MRSA reference strains, when compared to **4**. Finally, **5**, with a distinct scaffold among the others, was the only one showing a MIC to MSSA (200 μM) higher than that to MRSA (100 μM). Compound **5** is a natural product found in Casearia sylvestri, a medicinal plant broadly used in south America as anti-inflammatory, anticancer, antimicrobial, and antiulcer.^54–56^ Silva and co-workers described the antimicrobial activity against S. aureus from two metabolites derived from the gallic acid obtained from ethanolic extract from C. sylvestris leaves,^57^ and this information combined with the biological data reported here may help build a foundation to understanding the antimicrobial activity of this species. Also, compound **5** is an interesting hit because it already has pharmacokinetic properties determined. It has a high cell permeability; however, it may require formulation strategies due to degradation in the gastrointestinal tract.^50^ Other potential application for **5** is topical use since it will not need to be absorbed though the gastrointestinal tract.

The active compounds were assessed for cytotoxicity in Vero cells using the colorimetric MTT assay. Compound **2** is the least toxic (CC_50_ > 200 μM), while **3** and **4** showed higher cytotoxicity (CC_50_: 21.16 ± 8.89 and 19.03 ± 6.10 μM, respectively). In addition, compound **6** showed intermediate values (43.44 ± 4.66 μM) among the compounds, while **1** showed over five times lower CC_50_ values (33.75 ± 3.44 μM) in comparison to its analogue **2**. Lastly, the CC_50_ for **5** was not determined at the time. Altogether, **2** showed the highest SI for MSSA (>16), but unfortunately, it was inactive against resistant strains, and the other compounds’ MRSA strain’s SI ranged from 0.19 to 0.43, and E. coli’s SI was equal to 0.19. In combination, SI values of all compounds ranged from 0.19 to >16, following the lowest and highest CC_50_ values (**4** and **2**, respectively). The results of the cytotoxicity assays are summarized in Table 4.

Promising hit compounds preferably have SI values of at least ≥10.^49^ Compound **2** showed the highest value (SI > 16), and further changes could focus on lowering MIC values. The other five compounds, with SI values ranging from 0.19 to 0.43, could be theoretically improved by modifying minor substitutions. As examples, specifically for diarylamines derivatives, chloride and dichloride substituents, as observed in analogues from compound **4**, showed lower MIC values than their analogues.^25^ However, it is understood that most of their activity can be derived from toxicity or shared targets with the host cells. In this same work, the SI values of CPD22 and other lipophilic compounds are indeed somewhat similar (0.25 to 2.19) and were also active against S. aureus and MRSA strains.^25^ Although the cytotoxicity is a limiting factor to oral or intravenous administration of those compounds, they could still perform adequately in topical use,^58–61^ due to the direct absorption and in situ activity of lipophilic bioactive compounds. This administration route is relevant for the treatment of skin or cutaneous *Staphylococcus* infections.^62^ In addition, considering the hydrophobicity’s role in antibacterial activity,^52,53,63^ specially where substituted (iso)­flavonoids are concerned.^64^

Nonetheless, modifications to desired lipophilic structures can also be assessed for ultimately improving physicochemical properties and pharmacokinetics, and even enhancing antibacterial activity, including those against MRSA.^65^ Furthermore, specific chemical modifications toward more hydrophilic substituents or different positions (*e.g., meta* or *para*-substituted) could potentially lower the cellular toxicity *in vitro*,^66^ and thus, improve SI values.

Finally, we were able to determine confusion matrix-derived parameters (Supporting Table S3) for each model based on the experimental evaluation of the 62 compounds. In this regard, the MSSA and MRSA models had true positive rates equal to zero but acceptable true negative rates (0.62 and 0.50, respectively), indicating that those models are better at identifying substances that should not be tested than actual hits. In contrast, the model built from the SRI subset presented true positive rate equals to 50% (compound **6** assuming the 50 μM threshold specified in our methodology for MSSA and at least one MRSA). Despite the true positive rate of MSSA and MRSA models being lower in comparison to their true negative rates, our virtual screening performed equivalent or even better than other virtual screening campaigns reported in the literature.^67,68^ Furthermore, we analyzed the influence of the average value of probabilities on the confusion matrix metrics (true negative rate, accuracy, and balanced accuracy). As high as the cutoff is, the predictability of models (Supporting Figure S6) suggested that this parameter should be considered in future studies as a mandatory threshold for decision making.

Lastly, none of the six compound hits (**1**–**6**) was predicted to be active against methicillin-susceptible or resistant strains of S. aureus using the AntiBac-Pred Web server.^15^ This result reinforces the importance of having alternative tools for such prediction, since false negative and positive predictions are well-known limitations of *in silico* models. Therefore, relying on a single model/method could lead to a bottleneck of discoveries limiting the possibilities of candidates to be tested in the early stages of drug discovery and, ultimately, the diversity of available drugs in the clinical.

## Conclusions

Taken together, these results show the feasibility of the application of ML methods as viable options to support the virtual screening of compounds with antibacterial activity. In addition, with the large data curation and features for selection, the employment of this combination shows a promising strategy in the search and discovery of novel potential antibacterial compounds, which resulted in six hits, one with promising activity against S. aureus and five hits active against MRSA strains. As a perspective, we intend to investigate the activity of synthesis intermediates and analogues of the selected compounds against MSSA and MRSA and possibly evaluate them against other clinically relevant MDR bacteria, such as glycopeptide–intermediate S. aureus. The SRI data set comprising only the inactive and active compounds for both strains proved to be a suitable strategy for modeling both activities at the same time, excluding unnecessary information from the data set. Additionally, Fisher’s Weight, a simple fast approach to select features, performed well alongside more complex approaches. We hope that these data may help in the development of new promising compounds and toward further optimization of biological activity, aiming for the development of potential therapeutic agents for the treatment of bacterial infections, especially those caused by MRSA and other important Gram-positive resistant bacteria.

## Supplementary Material





## Data Availability

The data used in this work are available in a compressed folder named “Supporting data”. The molecular structures present in the three subsets (SRI, MSRA, and MSSA) are available in SDF format in the Supporting data folder (one SDF file per data set). All inputs, containing the descriptors (20 files per data set), used to train the ML models are available as well in the Supporting data folder in CSV files named the following organization: “dataset” + “variable section method” + “number of variables”. The KNIME workflow is also available in the GitHub repository available at https://github.com/MMLab-UFMG/ML-MRSA-MSSA Third-party software employed in the manuscript were as follows. R version 4.1.2 (https://www.r-project.org/) is an open-source free software. Discovery Studio (https://www.3ds.com/products/biovia/discovery-studio) is distributed under license. OMEGA version 3.1.1.2 (https://www.eyesopen.com/omega) and QUACPAC version 2.0.1.2 (https://www.eyesopen.com/quacpac) are distributed under the OpenEye Scientific license. The MASSA algorithm is implemented in the KNIME platform version 4.0.1, which is an open-source software.

## Notes

Strain distribution and classification according to the methicillin susceptibility; applicability domain analysis; confusion matrix and ML predictions; and *in vitro* assays results (PDF)
CHEMBL Datasets (ZIP)

## Notes

Professional language assistance service was performed by Pontual Traduções Ltd.a. (Londrina, Paraná, Brazil).

## Abbreviations

AMR: antimicrobial-resistant
ATCC: American type culture collection
AUC: area under the curve
CADD: computer-aided drug discovery
CC_50_: cytotoxic concentration of 50%
CDC: Centers of Disease Control and Prevention
CICC: China Center of Industrial Culture Collection
DSMZ: German Collection of Microorganisms and Cell Cultures
DT: decision tree
FDA: Food and Drug Administration
FN: false negatives
FP: false positives
FW: Fisher weight
HCA: hierarchical cluster analysis
HPLC: high-performance liquid chromatography
kNN: k-nearest neighbors
MCC: Matthews correlation coefficient
MDR: multidrug-resistant
MEM: Eagle’s minimum essential medium
MIC: minimal inhibitory concentration
ML: machine learning
MLP: multilayer perceptron
MRSA: methicillin-resistant S. aureus
MSSA: methicillin-susceptible S. aureus
MTCC: microbial type culture collection
MTT: 3-(4,5-dimethylthiazol-2-yl)-2,5-diphenyltetrazolium bromide
NB: naïve bayes
NMR: nuclear magnetic resonance
PBP: penicillin-binding protein
PCA: principal component analysis
QSAR: quantitative structure–activity relationship
RF: random forest
ROC: receiver operating characteristic curve
SI: selectivity index
TN: true negatives
TNR: true negative rate
TP: true positives
TPR: true positive rate
UPLC: ultraperformance liquid chromatography
WHO: World Health Organization
