# Supplementary material for: Machine Learning-Based Virtual Screening of Antibacterial Agents against Methicillin-Susceptible and Resistant Staphylococcus aureus
Source: J Chem Inf Model. 2024 Mar 4;64(6):1932–44. doi: 10.1021/acs.jcim.4c00087 (PMC12212627; doi:10.1021/acs.jcim.4c00087)
Supplement: Supplementary file 1 [file ci4c00087_si_001.pdf]

Supporting Information

Machine learning-based virtual screening of new antibacterial agents against methicillin-susceptible and resistant *Staphylococcus aureus*

Philipe Oliveira Fernandes[1], Anna Letícia Teotonio Dias[1], Valtair Severino dos Santos Júnior[1], Mateus Sá Magalhães Serafim[2], Yamara Viana Sousa[1], Gustavo Claro Monteiro[3], Isabel Duarte Coutinho[3], Marilia Valli[4], Marina Mol Sena Andrade Verzola[1], Flaviano Melo Ottoni[1], Rodrigo Maia de Pádua[1], Fernando Bombarda Oda[5], André Gonzaga dos Santos[5], Adriano Defini Andricopulo[4], Vanderlan da Silva Bolzani[3], Bruno Eduardo Fernandes Mota[6], Ricardo José Alves[1], Renata Barbosa de Oliveira[1], Thales Kronenberger[7,8], Vinícius Gonçalves Maltarollo[1*]

[1] Departamento de Produtos Farmacêuticos, Faculdade de Farmácia, Universidade Federal de Minas Gerais (UFMG), Belo Horizonte, Minas Gerais, 31.270-901, Brazil.

[2] Departamento de Microbiologia, Instituto de Ciências Biológicas, Universidade Federal de Minas Gerais (UFMG), Belo Horizonte, Minas Gerais, 31.270-901, Brazil.

[3] Departamento de Química Orgânica, Instituto de Química, Universidade Estadual Paulista (UNESP), Araraquara, São Paulo, 14.800-900, Brazil.

[4] Departamento de Física e Ciência Interdisciplinar, Instituto de Física, Universidade de São Paulo (USP), São Carlos, São Paulo, 13.563-120, Brazil.

[5] Departamento de Fármacos e Medicamentos, Faculdade de Ciências Farmacêuticas, Universidade Estadual Paulista (UNESP), Araraquara, 14.800-903, Brazil.

[6] Departamento de Análises Clínicas e Toxicológicas, Faculdade de Farmácia, Universidade Federal de Minas Gerais (UFMG), Belo Horizonte, Minas Gerais, 31.270-901, Brazil.

[7] Institute of Pharmacy, Pharmaceutical/Medicinal Chemistry and Tübingen Center for Academic Drug Discovery, Eberhard Karls University Tübingen, 72076 Tübingen, Germany.

[8] School of Pharmacy, Faculty of Health Sciences, University of Eastern Finland, 70211 Kuopio, Finland.

Supplementary Figures

Supplementary Figure S1. Relative frequency of the twenty more frequent *S. aureus* strains in the entries from ChEMBL database.

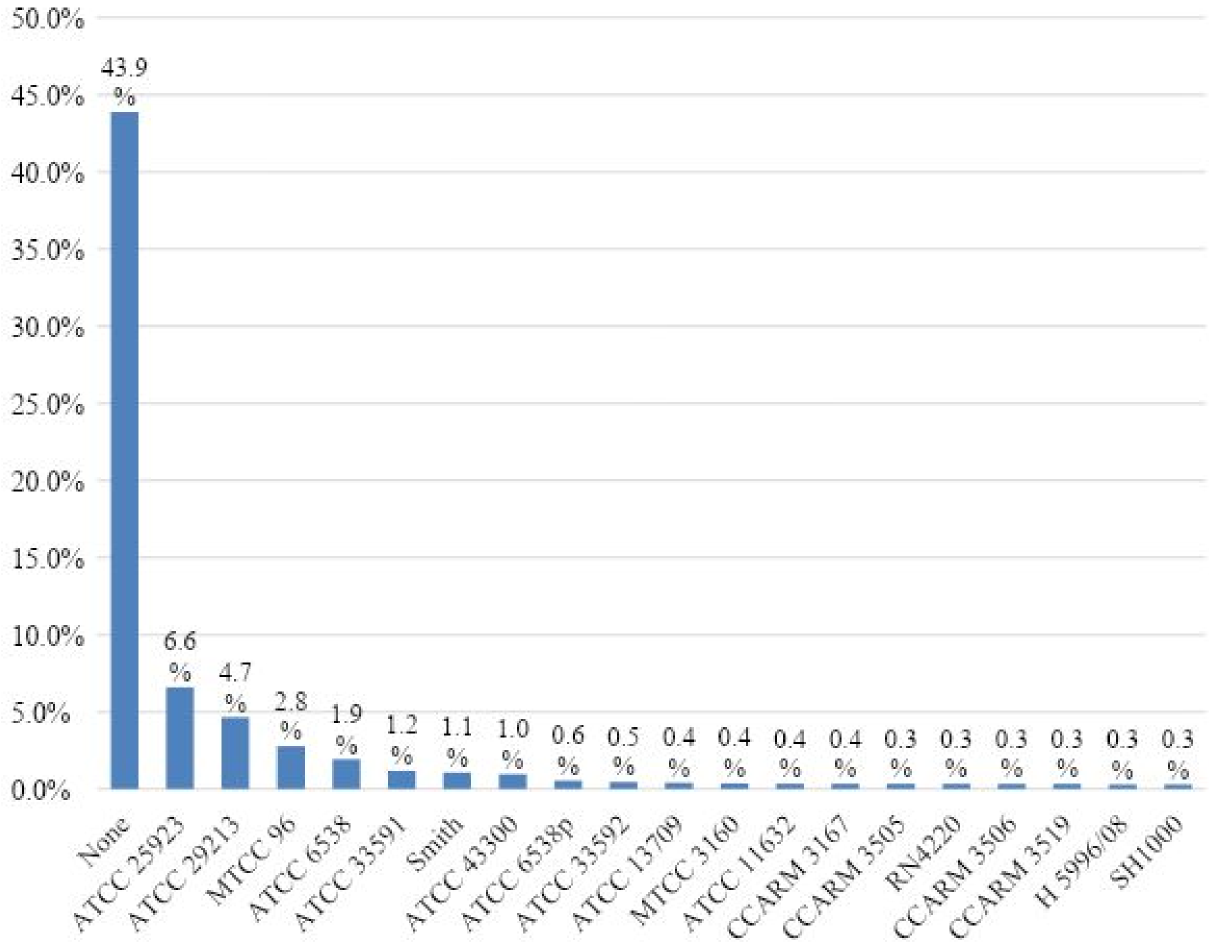

Supplementary Figure S2. Dissimilarity between the twenty variables selected for each feature selection method measured by the Jaccard's distance.

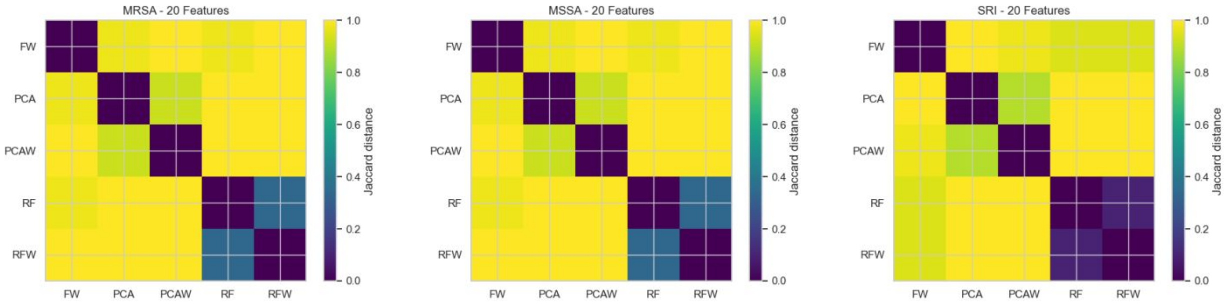

Supplementary Figure S3. t-SNE plot of the chemical space for the features selected by the Fisher's weight.

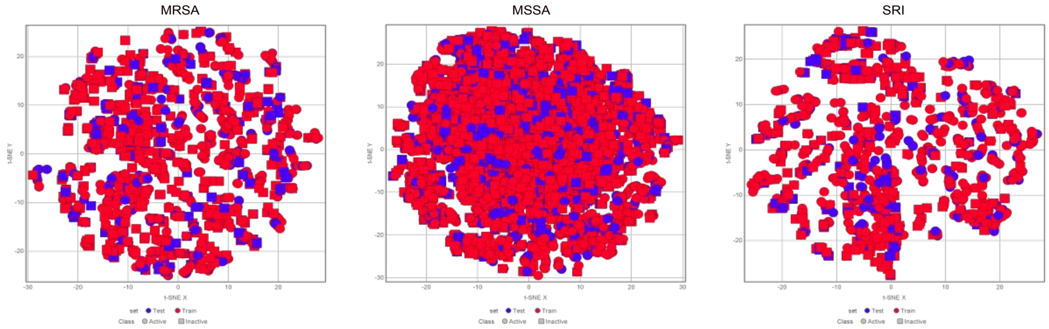

Supplementary Figure S4. Distribution between active (Class 1) and inactive (Class 0) in the features selected by the Fisher's Weight present in the SRI dataset.

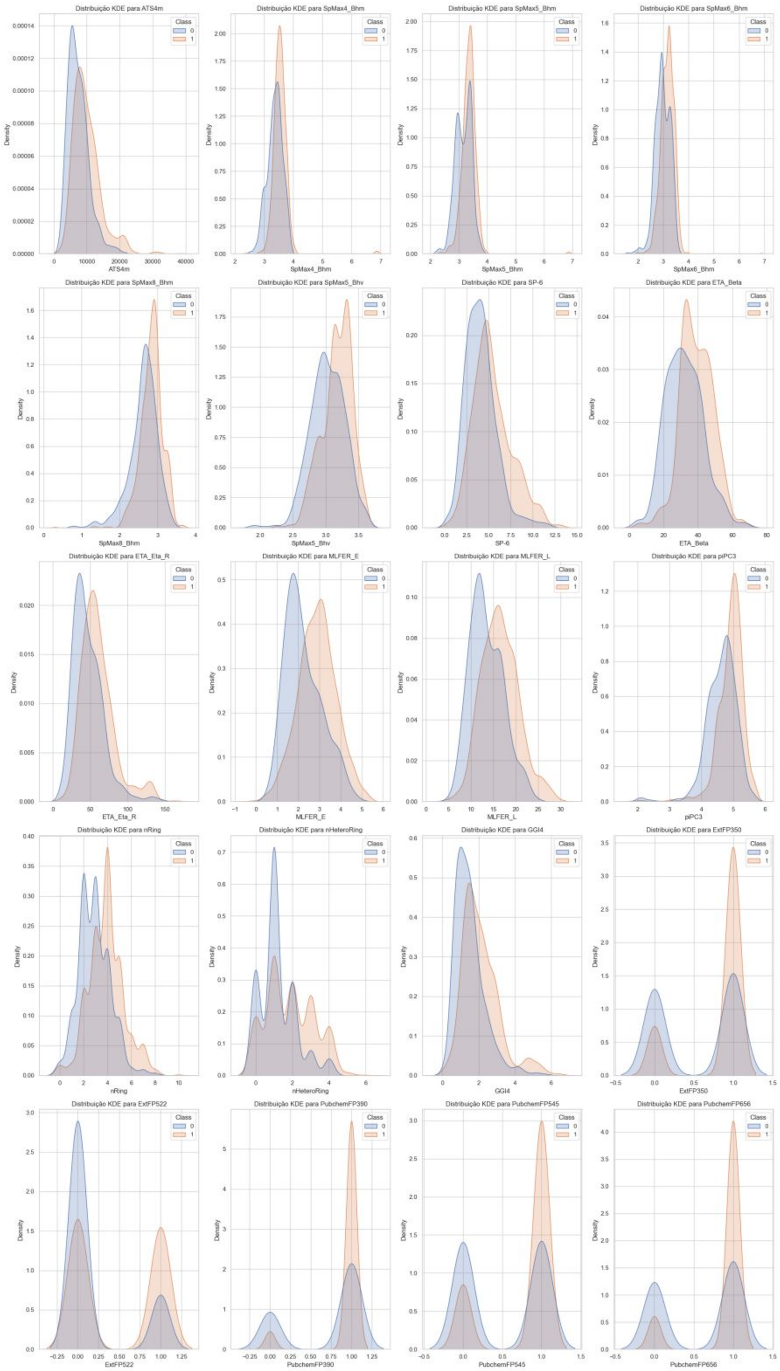

Supplementary Figure S5. Scatter plot from the applicability domain: a) Training (blue) and test (red) sets used to build the consensus models with 10 variables selected by Fisher's weight; b) Training set from the consensus models (blue) and virtual screening hits (red) using 10 variables selected by Fisher's weight; c) Training (blue) and test (red) sets used to build the consensus models with 15 variables selected by Fisher's weight used to build the consensus models; d) Training set from the consensus models (blue) and virtual screening hits (red) using 15 variables selected by Fisher's weight; e) Training (blue) and test (red) sets used to build the consensus models with 20 variables selected by Fisher's weight; f) Training set from the consensus models (blue) and virtual screening hits (red) using 20 variables selected by Fisher's weight g) Training (blue) and test (red) sets used to build the MRSA models with 20 variables selected by PCA; h) Training set from the MRSA models (blue) and virtual screening hits (red) using 20 variables selected by PCA; i) Training (blue) and test (red) sets used to build the MSSA models with 20 variables selected by Fisher's weight; j) Training set from the MSSA models (blue) and virtual screening hits (red) using 20 variables selected by Fisher's weight; k) Training (blue) and test (red) sets used to build the MSSA models with 20 variables selected by PCA; l) Training set from the MSSA models (blue) and virtual screening hits (red) using 20 variables selected by PCA.

a)

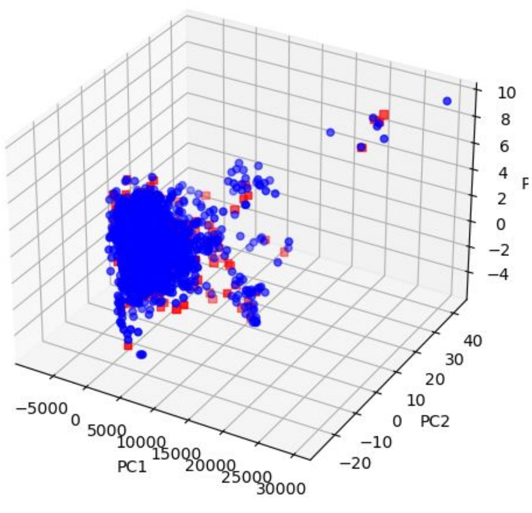

b)

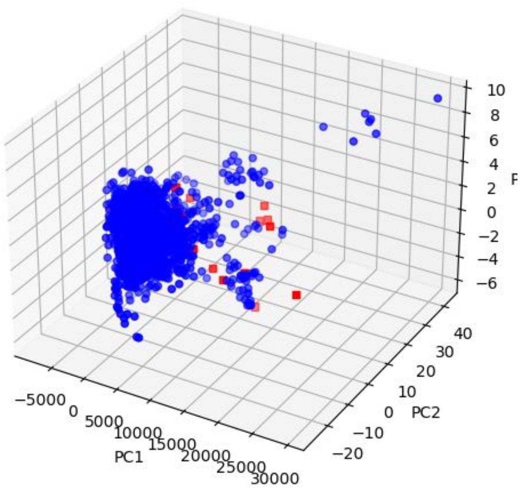

c)

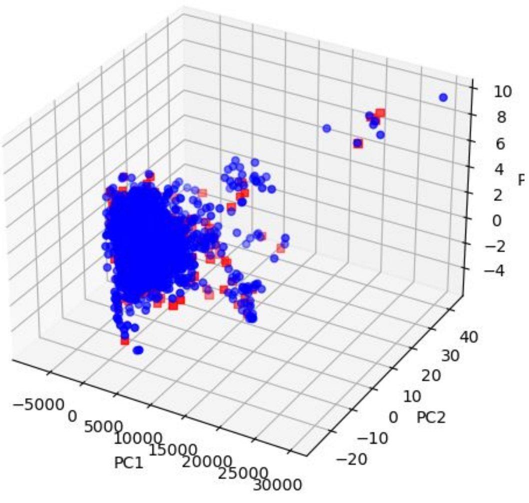
PC1
PC2
-5000 0 5000 10000 15000 20000 25000 30000
-20 -10 0 10 20 30 40
10 8 6 4 2 0 -2 -4

d)

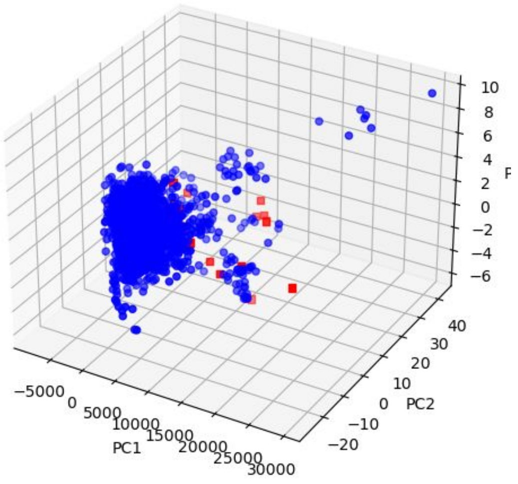
PC1
PC2
-5000 0 5000 10000 15000 20000 25000 30000
-20 -10 0 10 20 30 40
10 8 6 4 2 0 -2 -4 -6

e)

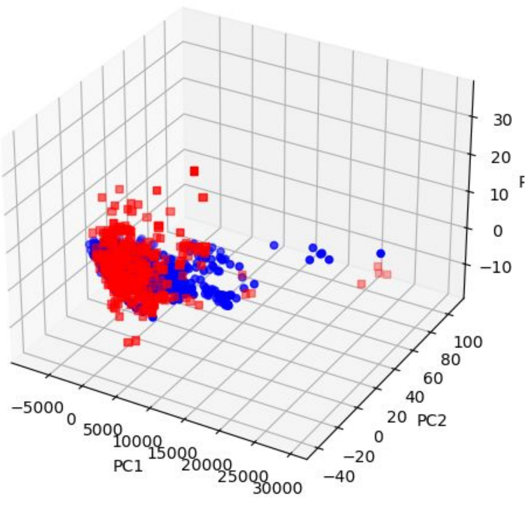
PC1
PC2
-5000 0 5000 10000 15000 20000 25000 30000
-40 -20 0 20 40 60 80 100
30 20 10 0 -10

f)

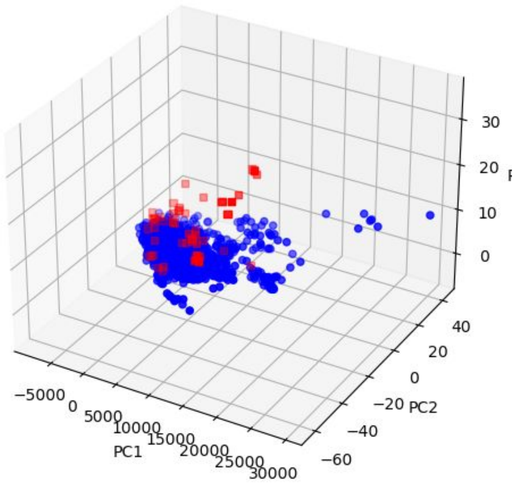
PC1
PC2
-5000 0 5000 10000 15000 20000 25000 30000
-60 -40 -20 0 20 40
30 20 10 0

g)

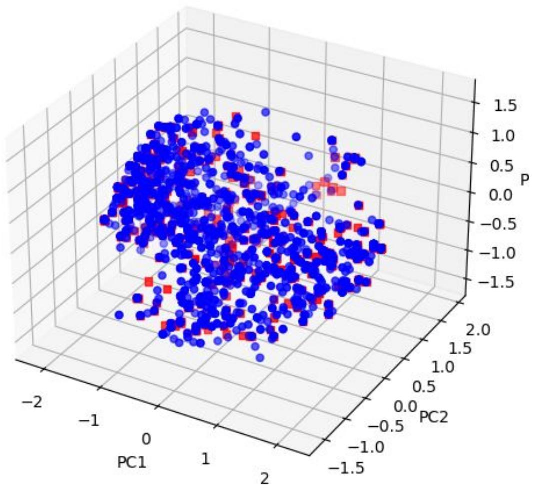
PC1
PC2
P
−2 −1 0 1 2
−1.5 −1.0 −0.5 0.0 0.5 1.0 1.5 2.0
−1.5 −1.0 −0.5 0.0 0.5 1.0 1.5

h)

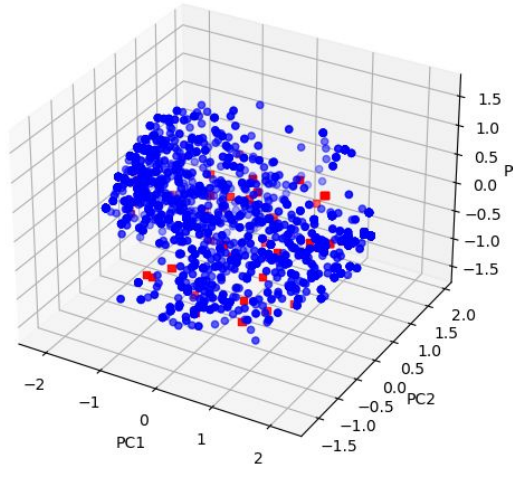
PC1
PC2
P
−2 −1 0 1 2
−1.5 −1.0 −0.5 0.0 0.5 1.0 1.5 2.0
−1.5 −1.0 −0.5 0.0 0.5 1.0 1.5

i)

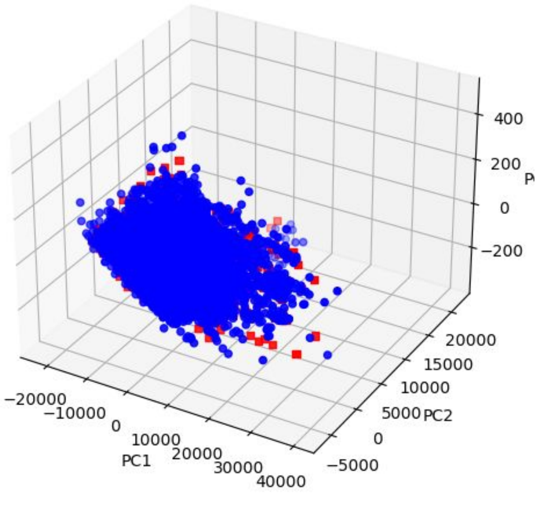
PC1
PC2
P
−20000 −10000 0 10000 20000 30000 40000
−5000 0 5000 10000 15000 20000
−200 0 200 400

j)

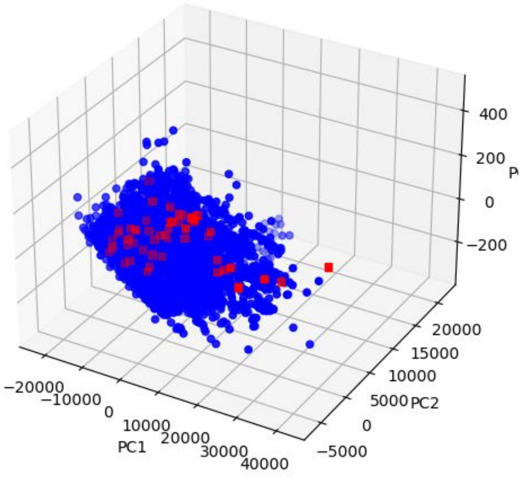
PC1
PC2
P
−20000 −10000 0 10000 20000 30000 40000
−5000 0 5000 10000 15000 20000
−200 0 200 400

k)

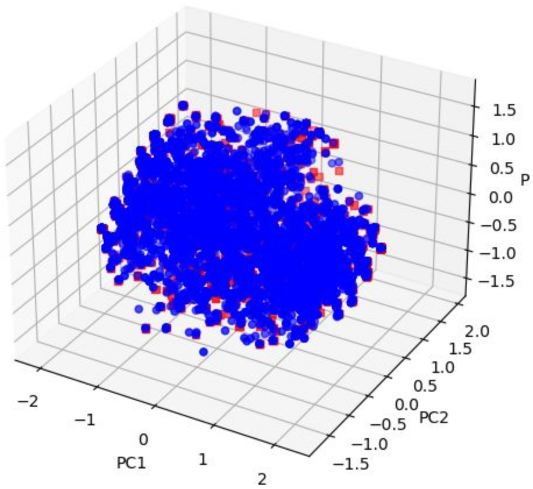

l)

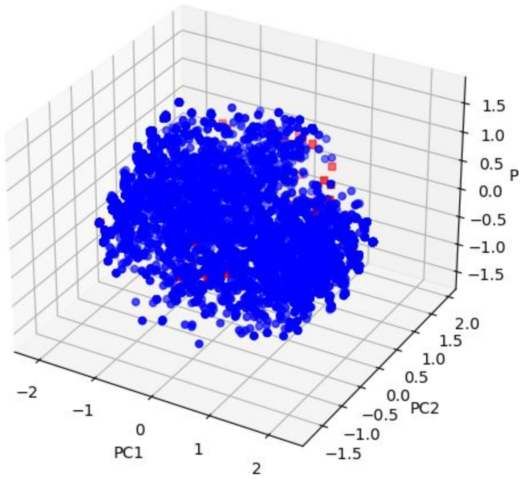

Supplementary Figure S6. Different classification thresholds influence in the models predictivity compared to experimental validation results of tested hits.

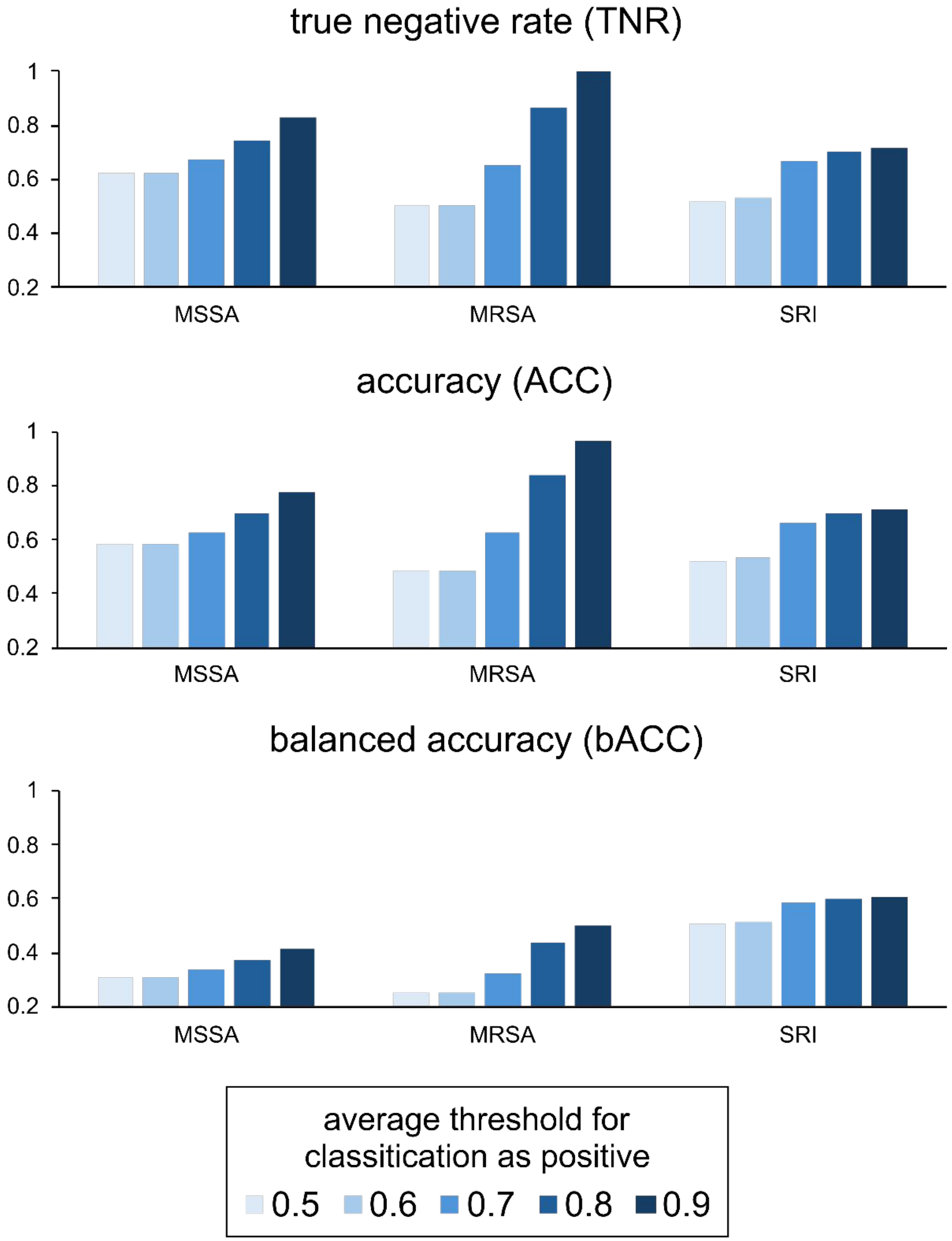

Supplementary Tables

Supplementary Table S1. Classification of the *S. aureus* strains found in the ChEMBL database entries as methicillin-resistant *S. aureus* (MRSA) or methicillin-susceptible *S. aureus* (MSSA).

Strain	Class	Strain	Class
ATCC 25923	MSSA	NCIM 2079/ATCC 6538P	MSSA
ATCC 29213	MSSA	ATCC 101 29213	MSSA
MTCC 96	MSSA	ATCC6538	MSSA
ATCC 6538	MSSA	FDA 209-P	MSSA
Smith	MSSA	NCIM 2079; ATCC6538P	MSSA
ATCC 6538p	MSSA	ATCC 33591	MRSA
ATCC 13709	MSSA	ATCC 43300	MRSA
MTCC 3160	MSSA	ATCC 33592	MRSA
ATCC 11632	MSSA	CCARM 3167	MRSA
SH1000	MSSA	RN4220	MRSA
KCTC 503	MSSA	CCARM 3506	MRSA
ATCC 9144	MSSA	H 5996/08	MRSA
CCM 4516/08	MSSA	CBD-635	MRSA
NCIM 5021	MSSA	ATCC 700699	MRSA
8325-4	MSSA	N315	MRSA

209P	MSSA	USA300	MRSA
KCTC 209	MSSA	SA1199B	MRSA
MTCC 1430	MSSA	ATCC BAA-44	MRSA
MS5935	MSSA	Mu50	MRSA
ATCC 35556	MSSA	NCIM 2079	MRSA
ATCC 12600	MSSA	Mu50omega	MRSA
Oxford	MSSA	OIT971	MRSA
ATTC 25923	MSSA	SR3637	MRSA
NCTC 4163	MSSA	XU212	MRSA
MLS16 MTCC 2940	MSSA	OM584	MRSA
DSM 1104	MSSA	134/93	MRSA
MLS-16 MTCC 2940	MSSA	BAA-44	MRSA
29213	MSSA	SCV	MRSA
NCTC 7447	MSSA	UAMS-1	MRSA
SG511	MSSA	NRS271	MRSA
209-P	MSSA	1094	MRSA
IFO 3060	MSSA	ATCC 43866	MRSA
CIP 4.83	MSSA	OM481	MRSA
ATCC 29737	MSSA	N 315	MRSA
MB2865	MSSA	MRSA 4591	MRSA
E19977	MSSA	RN 4220	MRSA

ATCC25923	MSSA	NRS119	MRSA
CMCC 26003	MSSA	COL	MRSA
MN8	MSSA	KMP9	MRSA
MTCC 737	MSSA	UAMS1	MRSA
RDN1	MSSA	DSM 50128509	MRSA
MTCC 096	MSSA	EMRSA-15	MRSA
SR20549	MSSA	ATCC 700787	MRSA
Rosenbach 209	MSSA	ATCC 700698	MRSA
DM4001	MSSA	BAA 44	MRSA
ARC516	MSSA	SA-1199B	MRSA
CCM 3953	MSSA	EMRSA-16	MRSA
Smith ATCC 13709	MSSA	Mu3	MRSA
209P JC1	MSSA	ATCC43300	MRSA
MTCC 7443	MSSA	NRS 70	MRSA
SG 511	MSSA	ATCC BAA-39	MRSA
CB190	MSSA	USA200	MRSA
NCTC 6571	MSSA	USA500	MRSA
25923	MSSA	NRS70	MRSA
UC76	MSSA	USA100	MRSA
Newman	MSSA	USA700	MRSA
ATCC 12598	MSSA	NRS1	MRSA

ATCC 25293	MSSA	ATCC700699	MRSA
CECT 86	MSSA	BAA-1707	MRSA
209	MSSA	isolate USA300	MRSA
KCTC 1621	MSSA	USA300 SF8300	MRSA
UC-76 SA-1	MSSA	USA-300	MRSA
MTCC-96	MSSA	BAA-39	MRSA
ATCC-25923	MSSA	43300	MRSA
NRS72	MSSA	ATCC BAA-1720	MRSA
UC-76	MSSA	CA ATCC BAA-1680	MRSA
ATCC 13709 Smith isolate SA100	MSSA	isolate MU50	MRSA
Seattle 1945 ATCC 25923	MSSA	CCARM 3089	MRSA
FDA209P	MSSA	CCARM 3090	MRSA
MSSA 22	MSSA	COL-S	MRSA
6538P	MSSA	MRSA 1094	MRSA
ATCC6538P	MSSA	NRS18	MRSA
209 P(FDA)	MSSA	ATCC 700789	MRSA
CECT 86 ATCC 12600	MSSA	ATCC BAA-1556	MRSA
FDA 209P	MSSA	ATCC BAA-1680	MRSA
Smith OC 4172	MSSA	ATCC BAA-1685	MRSA
Smith diffuse	MSSA	ATCC BAA-1753	MRSA

ATCC 13709 (Smith)	MSSA	ATCC BAA-811	MRSA
ATCC29213	MSSA	MW2 serotype USA400	MRSA
ATTC-25923	MSSA		

Supplementary Table S2. Descriptor list used to train the final models.

SRI dataset FW 20 features	MRSA dataset PCA 20 features	MSSA dataset FW 20 features	MSSA dataset PCA 20 features
ATS4m	ExtFP82	ATS3m	ExtFP26
SpMax4_Bhm	ExtFP220	ATS2v	ExtFP220
SpMax5_Bhm	ExtFP374	ATS0s	ExtFP328
SpMax6_Bhm	ExtFP411	ATSC0c	ExtFP406
SpMax8_Bhm	ExtFP420	ATSC0i	ExtFP420
SpMax5_Bhv	ExtFP598	SPC-4	ExtFP508
SP-6	ExtFP953	nHBa	ExtFP582
ETA_Beta	ExtFP1013	nHBAcc2	ExtFP623
ETA_Eta_R	MACCSFP95	nHBAcc_Lipinski	ExtFP686
MLFER_E	MACCSFP110	IC2	ExtFP717
MLFER_L	MACCSFP120	IC3	ExtFP791
piPC3	MACCSFP136	MPC4	ExtFP823
nRing	PubchemFP439	nHeteroRing	ExtFP956
nHeteroRing	PubchemFP645	GGI1	ExtFP1013
GGI4	SubFP88	WTPT-3	EStateFP30
ExtFP350	KRFP3295	AD2D173	MACCSFP97
ExtFP522	KRFP3455	AD2D179	KRFP3740
PubchemFP390	KRFP3773	SubFPC295	KRFP3750
PubchemFP545	AD2D404	KRFPC3750	KRFP3773
PubchemFP656	AD2D704	APC2D5_C_F	AD2D404

Supplementary Table S3. Percentage of samples inside the applicability domain for the test set and hits from the virtual screening.

Endpoint	Algorithm	Descriptors		Percentage of samples inside the domain	
		Selection Method	Number of variables	Test set	Virtual screening
Consensus	kNNw	FW	10	100	100*
	DT		15	99	100*
	kNN		20	100	100*
MRSA	kNN/MLP/SVM	PCA	20	100	100
MSSA	kNN/kNNw	FW	20	100	100*
	DT	PCA	20	99	98

DT: decision tree; FW: Fisher's weight; kNN: k-nearest neighbors; kNNw: weighted k-nearest neighbors; PCA: principal component analysis.

* Compound S24 was disregarded from the applicability domain assessment due to errors in the descriptors calculation.

Supplementary Table S4. Cumulative active predictions by MRSA, MSSA, and Consensus models for the inactive compounds experimented validated thought percentual of inhibition at 100 uM.

ID	Source	Structure	SRI predictions		MRSA predictions		MSSA predictions		Percentage of growth inhibition				
			Sum	Prob	Sum	Prob	Sum	Prob	MSSA (ATCC 29123)	MRSA (ATCC 43300)	MRSA (5749)	MRSA (6154)	*E. coli* (ATCC 35218)
S01	BraCoLi	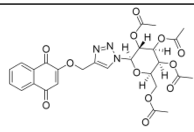	3	0.982	3	0.679	1	0.222	0	0	0	0	0
S02	BraCoLi	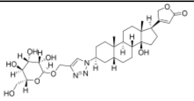	3	0.982	3	0.679	1	0.222	0	0	0	0	0
S03	BraCoLi	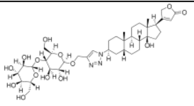	3	0.982	3	0.763	2	0.708	0	0	0	0	0
S04	BraCoLi	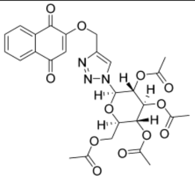	3	0.982	3	0.891	3	0.889	0	0	0	0	0
S05	BraCoLi	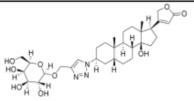	3	0.982	3	0.891	3	0.889	0	0	0	0	0
S06	BraCoLi	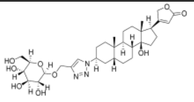	3	0.982	3	0.777	3	0.963	0	0	0	0	0
S07	BraCoLi	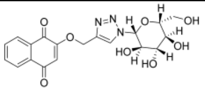	3	0.982	3	0.777	3	0.963	0	0	0	0	0

S08	BraCoLi	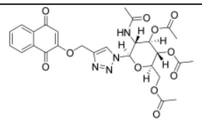	3	0.982	3	0.777	3	0.963	0	0	0	0	0
S09	BraCoLi	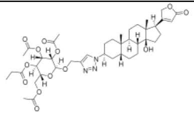	3	0.982	3	0.777	1	0.296	0	0	0	0	0
S10	BraCoLi	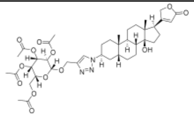	3	0.957	0	0.298	1	0.296	0	0	0	0	0
S11	BraCoLi	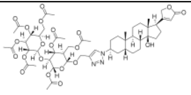	3	0.833	3	0.891	3	0.889	0	0	0	0	0
S12	BraCoLi	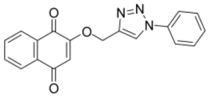	3	1.000	3	0.684	1	0.222	0	0	0	0	0
S13	BraCoLi	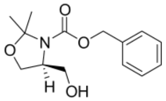	0	0.067	1	0.471	1	0.333	0	0	0	0	0
S14	BraCoLi	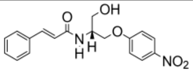	1	0.333	3	0.801	0	0.078	0	0	0	0	0
S15	BraCoLi	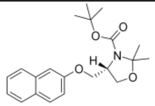	0	0.042	3	0.690	1	0.333	0	0	0	0	0
S16	BraCoLi	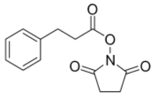	0	0.007	1	0.426	0	0.000	0	0	0	0	0
S17	BraCoLi	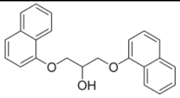	2	0.718	3	0.707	2	0.750	0	0	0	0	0
S18	BraCoLi	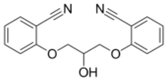	2	0.556	3	0.707	0	0.067	0	0	0	0	0

S19	BraCoLi	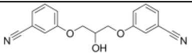	2	0.714	3	0.707	1	0.203	0	0	0	0	0
S20	BraCoLi	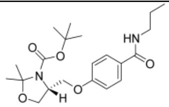	0	0.000	3	0.780	0	0.000	0	0	0	0	0
S21	BraCoLi	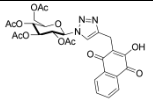	1	0.417	3	0.679	1	0.222	0	0	0	0	0
S22	BraCoLi	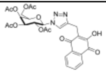	1	0.417	3	0.679	1	0.222	0	0	0	0	0
S23	BraCoLi	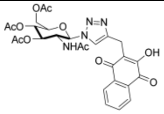	3	0.982	3	0.763	3	1.000	0	0	0	0	0
S24	BraCoLi	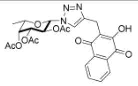	0*	0*	0*	0.187	0*	0*	0	0	0	0	0
S25	BraCoLi	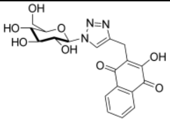	2	0.645	1	0.304	0	0.067	0	0	0	0	0
S26	BraCoLi	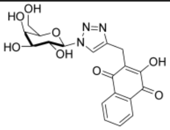	2	0.645	1	0.304	0	0.067	0	0	0	0	0
S27	BraCoLi	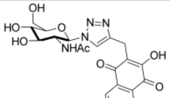	3	0.978	1	0.353	1	0.333	0	0	0	0	0
S28	BraCoLi	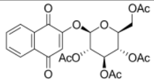	0	0.078	1	0.390	3	0.857	0	0	0	0	0
S29	BraCoLi	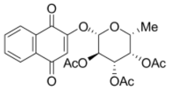	1	0.411	1	0.390	1	0.250	0	0	0	0	0

S30	BraCoLi	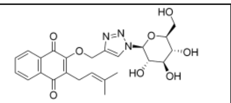	2	0.645	0	0.298	1	0.296	0	0	0	0	0
S31	BraCoLi	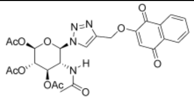	2	0.649	3	0.763	0	0.042	0	0	0	0	0
S32	BraCoLi	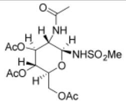	3	0.988	1	0.351	0	0.111	0	0	0	0	0
S33	BraCoLi	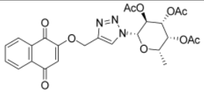	2	0.655	3	0.679	0	0.053	0	0	0	0	0
S34	BraCoLi	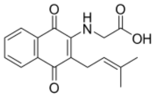	0	0.000	0	0.137	2	0.667	0	0	0	0	0
S35	BraCoLi	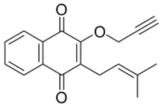	0	0.000	0	0.185	2	0.689	0	0	0	0	0
S36	BraCoLi	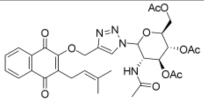	3	0.982	3	0.763	2	0.708	0	0	0	0	0
S37	BraCoLi	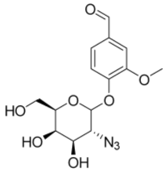	0	0.000	3	0.834	1	0.229	0	0	0	0	0
S38	BraCoLi	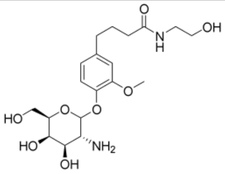	2	0.655	1	0.640	1	0.222	0	0	0	0	0

S39	BraCoLi	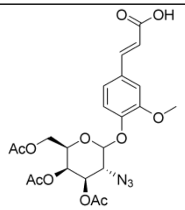	0	0.078	0	0.292	1	0.229	0	0	0	0	0
S40	BraCoLi	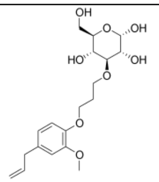	0	0.100	3	0.838	3	0.857	0	0	0	0	0
S41	BraCoLi	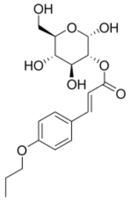	0	0.000	0	0.090	2	0.697	0	0	0	0	0
S42	BraCoLi	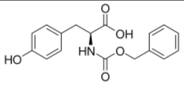	1	0.333	1	0.299	1	0.267	0	0	0	0	0
S43	BraCoLi	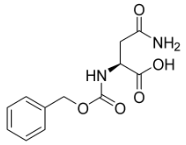	0	0.025	0	0.282	0	0.095	0	0	0	0	0
S44	BraCoLi	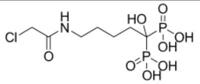	1	0.381	1	0.401	0	0.087	0	0	0	0	0
S45	BraCoLi	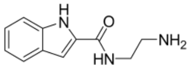	0	0.007	0	0.278	1	0.250	0	0	0	0	0
S46	BraCoLi	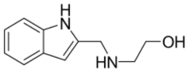	0	0.007	0	0.165	1	0.271	0	0	0	0	0

S47	BraCoLi	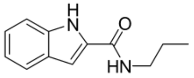	0	0.007	0	0.301	0	0.095	0	0	0	0	0
S48	BraCoLi	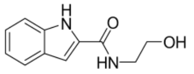	0	0.007	0	0.324	0	0.077	0	0	0	0	0
S49	BraCoLi	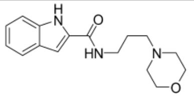	0	0.000	0	0.151	1	0.222	0	0	0	0	0
S50	NuBBE	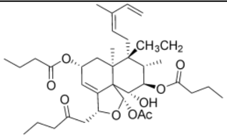	1	0.417	0	0.262	3	0.917	0	0	0	0	0
S51	NuBBE	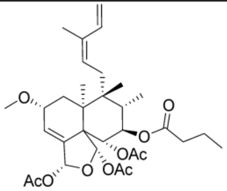	1	0.417	0	0.190	3	0.917	0	0	0	0	0
S52	NuBBE	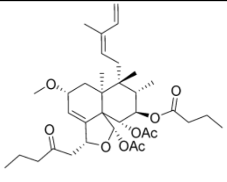	1	0.417	0	0.190	2	0.708	0	0	0	0	0
S53	NuBBE	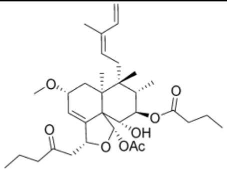	0	0.083	0	0.262	3	0.933	0	0	0	0	0
S54	NuBBE	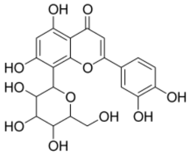	1	0.306	0	0.126	0	0.074	0	0	0	0	0

S55	*in house (trametinib)*	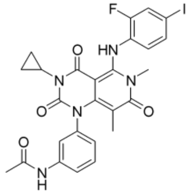	2	0.649	3	0.879	3	0.944	0	0	0	0	0
S56	*in house (Selumetinib)*	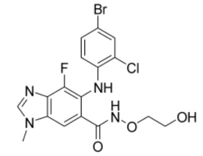	3	1.000	3	0.715	3	0.944	0	0	0	0	0

* Compound S24 suffered errors in the descriptors calculation.

Supplementary Table S5. Confusion matrix for the MSSA, MRSA, and SRI ~~consensus~~ models. A positive prediction was considered when at least two models predicted the compound as active for each endpoint.

**MSSA models**			**MRSA models**			**~~Consensus~~ SRI models**		
	active (pred)	*inactive (pred)*		*active (pred)*	*inactive (pred)*		*active (pred)*	*inactive (pred)*
active (exp)	0	4	*active (exp)*	0	2	*active (exp)*	1	1
inactive (exp)	22	36	*inactive (exp)*	30	30	*inactive (exp)*	29	31

**TPR**	0.00	**TPR**	0.00	**TPR**	0.50
**TNR**	0.62	**TNR**	0.50	**TNR**	0.52
**FPR**	0.38	**FPR**	0.50	**FPR**	0.48
**FNR**	1.00	**FNR**	1.00	**FNR**	0.50
**ACC**	0.58	**ACC**	0.48	**ACC**	0.52
**bACC**	0.31	**bACC**	0.25	**bACC**	0.51

ACC: accuracy; bACC: balanced accuracy; FNR: false negative rate; FPR: false positive rate; TNR: true negative rate; TPR: true positive rate.

Characterization Data

1. 1,3-bis(4-methylphenoxy)propano-2-aminium chloride (4)

1.1 Infrared spectrum of (4)

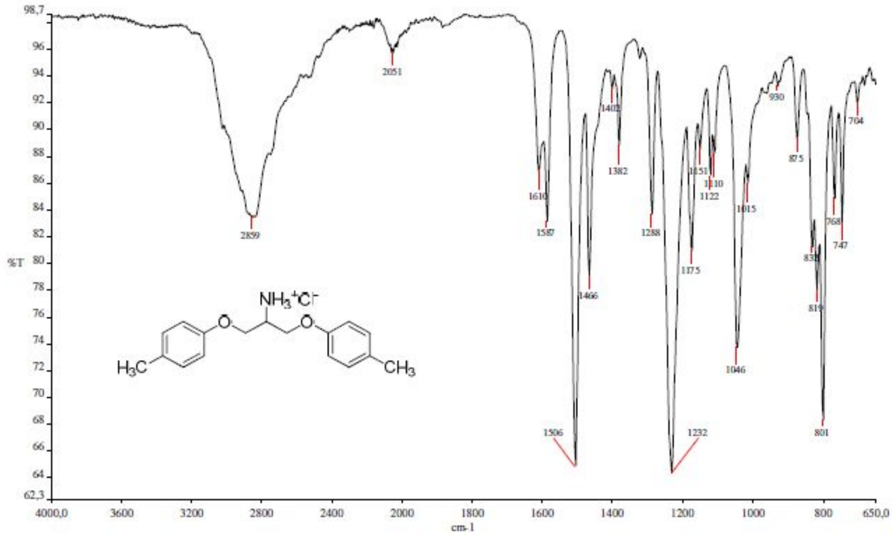

1.2 ^{1}H NMR spectrum of (4) ($CDCl_3$, 200 MHz)

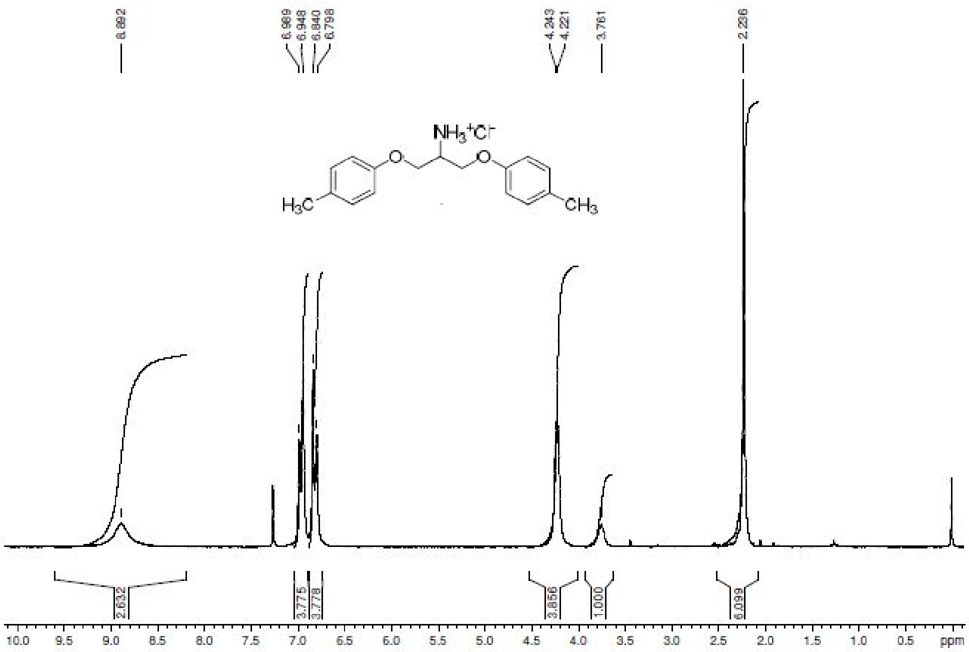

1.3 ^{13}C and DEPT135 NMR spectra of (4) ($CDCl_3$, 50 MHz)

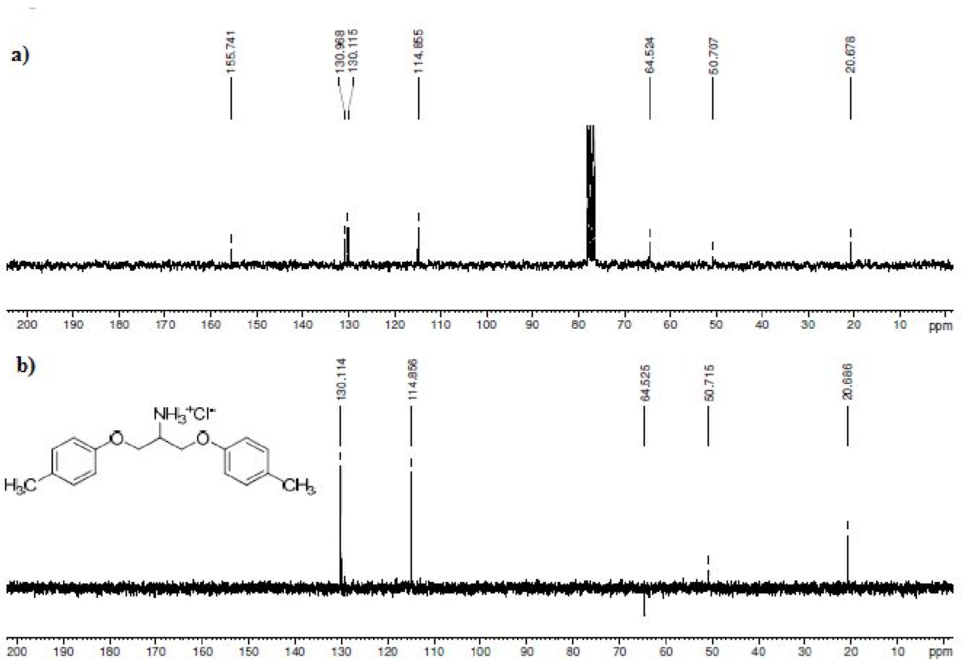

2. N,N'-(oxybis(propane-3,1-diyl))-bis-(5-(4-bromophenyl)furan-2-carboxamide) (6)

This product was obtained unexpectedly during an attempt to synthesize an azido derivative from a nucleophilic substitution reaction of a mesyl derivative with sodium azide, as described as follows: In a round-bottom flask, containing 0.2 g (0.5 mmol) of 3-(5-(4-bromophenyl)furan-2-carboxamido)propyl-methanesulfonate solubilized in a 3:1 THF/H_2O mixture, 0.160 g (2.5 mmol) of NaN_3 was added. The reaction was maintained under magnetic stirring at room temperature for approximately 4 hours, when the completion of the reaction was determined by TLC (eluent: ethyl acetate; stain: CAM). The crude product was purified by silica gel column chromatography (eluent: AcOEt), leading to obtaining dimer **6** in 20% yield.

Melting point: 87.0-89.0 ºC; HRMS (*m/z*) 631.0264; 629.0279 $[M^{+}H]^{+}$, calcd 631.0261; 629.0281 $C_{28}H_{27}Br_2N_2O_5$.

2.1 Infrared spectrum of (6)

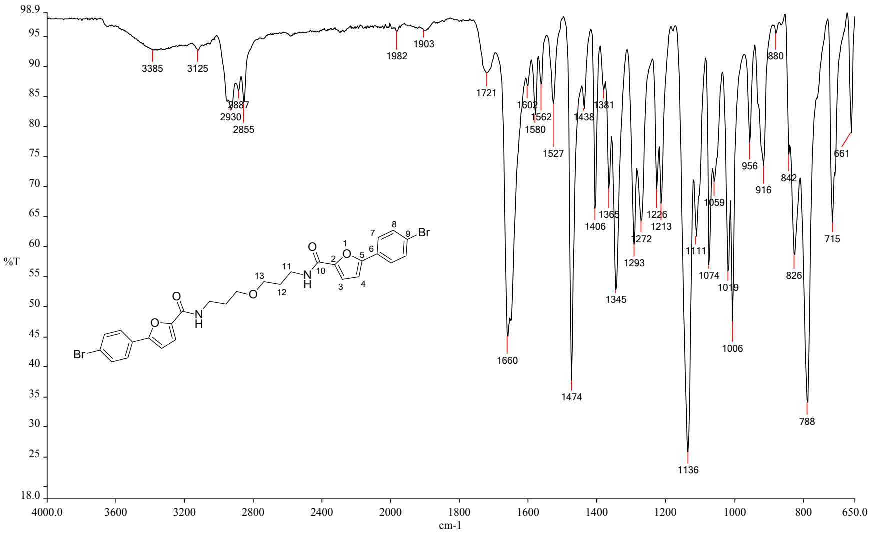

2.2 ^{1}H NMR spectrum of (6) (DMSO-*d*6, 400 MHz)

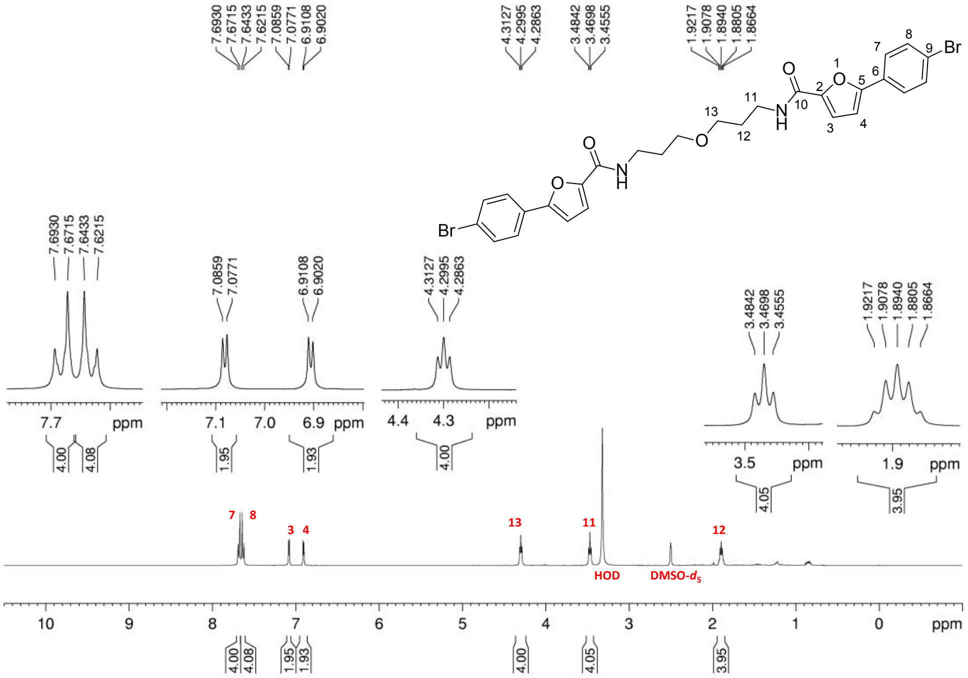

2.3 ^{13}C and DEPT135 NMR spectra of (6) (DMSO-d_6, 100 MHz)

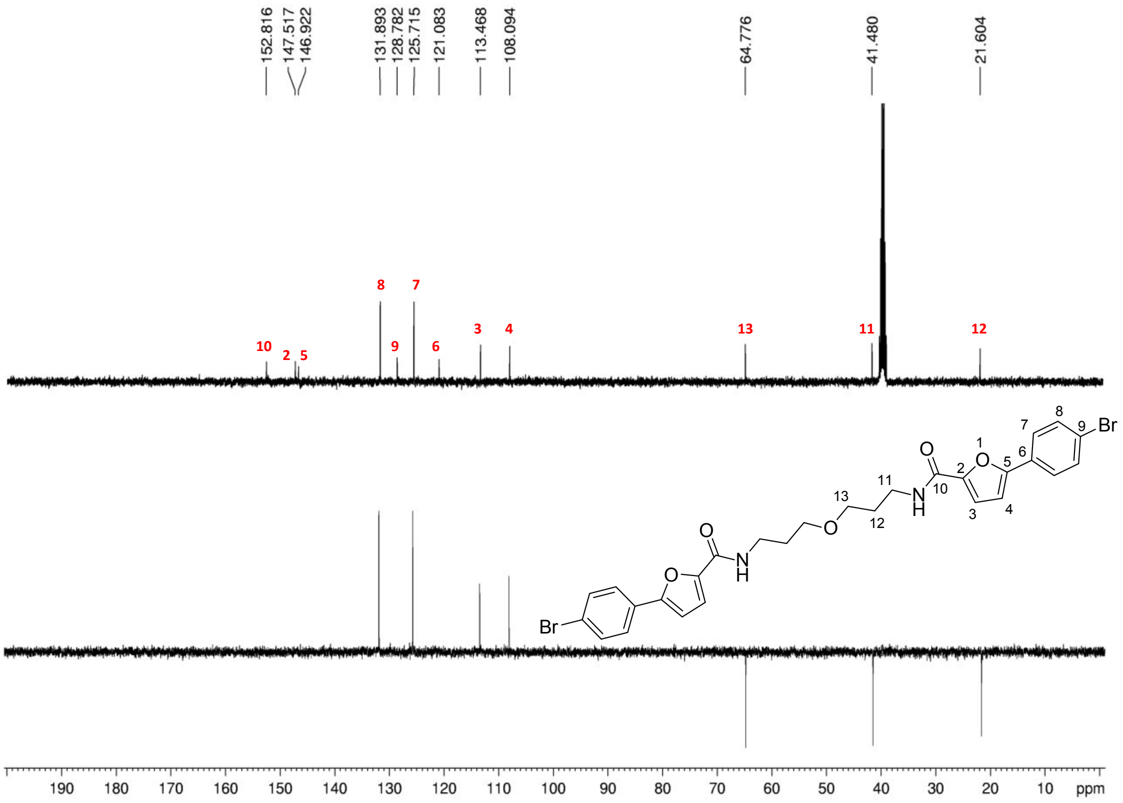

2.4 HRMS (ESI) spectrum of (**6**)

HRMS (*m/z*) 631.0264; 629.0279 [M$^+$H]$^+$, calcd 631.0261; 629.0281 $C_{28}H_{27}Br_2N_2O_5$.

Compound Spectrum Report

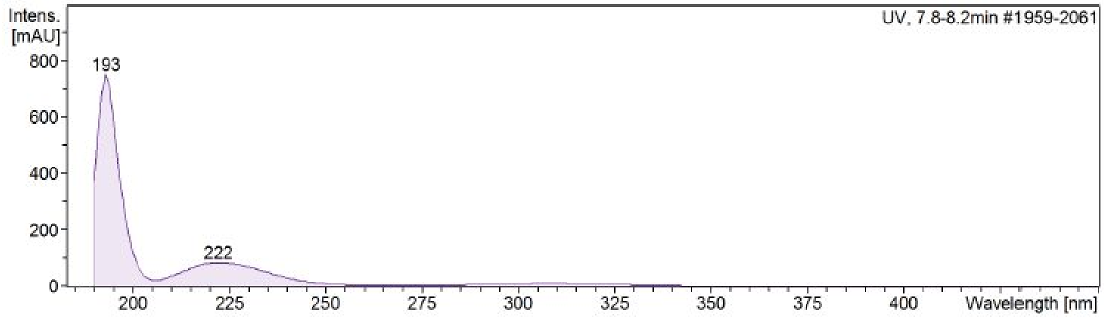

#	Wavelength	Intensity
0	193	747.1
1	222	84.6

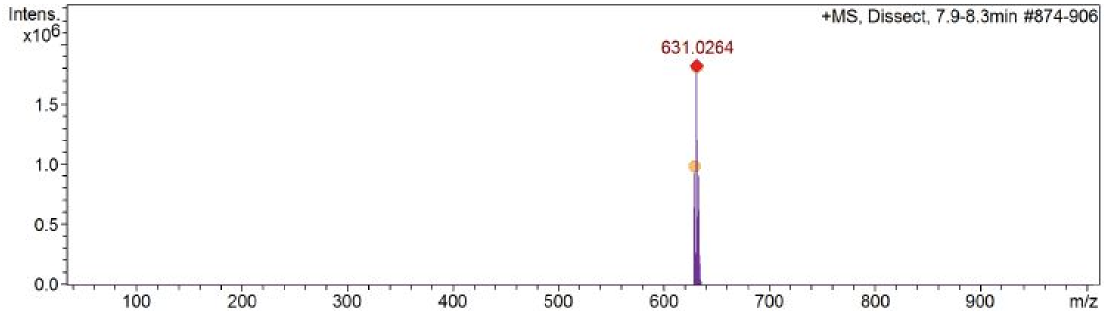

Meas. m/z	#	Ion Formula	Adduct	Sum Formula	Score	mSigma	m/z	err [mDa]	err [ppm]	rdb	e⁻ Conf	N-Rule
629.0279	1	C28H27Br2N2O5	M+H	C28H26Br2N2O5	100.00	14.9	629.0281	0.2	0.3	15.5	even	ok
	2	C16H31Br2N4O12	M+H	C16H30Br2N4O12	11.03	57.0	629.0300	2.0	3.3	2.5	even	ok
	3	C41H14BrN2O	M+H	C41H13BrN2O	0.00	269.7	629.0284	0.5	0.8	35.5	even	ok
	4	C28H22BrO12	M+H	C28H21BrO12	0.00	284.4	629.0289	1.0	1.6	17.5	even	ok
	5	C23H22BrN2O14	M+H	C23H21BrN2O14	0.00	287.0	629.0249	-3.0	-4.8	13.5	even	ok
	1	C31H28Br2NaO3	M+Na	C31H28Br2O3	100.00	17.8	629.0297	1.8	2.9	16.5	even	ok
	2	C26H28Br2N2NaO5	M+Na	C26H28Br2N2O5	73.75	17.9	629.0257	-2.2	-3.5	12.5	even	ok
	3	C26H23BrNaO12	M+Na	C26H23BrO12	0.00	286.6	629.0265	-1.4	-2.3	14.5	even	ok
	4	C27H19BrN4NaO8	M+Na	C27H19BrN4O8	0.00	289.1	629.0278	-0.1	-0.1	19.5	even	ok
	5	C39H15BrN2NaO	M+Na	C39H15BrN2O	0.00	290.2	629.0260	-1.9	-3.1	32.5	even	ok
	1	C16H31Br2N4O12	2M+H	C8H15BrN2O6	11.03	57.0	629.0300	2.0	3.3	2.5	even	ok
	2	C36H9N2O10	2M+H	C18H4NO5	0.00	545.3	629.0252	-2.8	-4.4	33.5	even	ok
	3	C42H5N4O4	2M+H	C21H2N2O2	0.00	546.9	629.0305	2.6	4.1	42.5	even	ok
	1	C14H22NaO26	2M+Na	C7H11O13	0.00	546.2	629.0292	1.2	1.9	3.5	even	ok
	2	C40H6N4NaO4	2M+Na	C20H3N2O2	0.00	548.2	629.0281	0.2	0.3	39.5	even	ok
631.0264	1	C31H20BrO10	M+H	C31H19BrO10	68.44	213.8	631.0234	-2.9	-4.6	21.5	even	ok
	2	C44H7O6	M+H	C44H6O6	40.08	218.1	631.0237	-2.6	-4.2	41.5	even	ok
	3	C45H3N4O2	M+H	C45H2N4O2	82.48	221.3	631.0251	-1.3	-2.1	46.5	even	ok
	4	C32H11N2O13	M+H	C32H10N2O13	100.00	222.4	631.0256	-0.8	-1.2	28.5	even	ok
	5	C50H3N2	M+H	C50H2N2	24.20	222.7	631.0291	2.7	4.3	50.5	even	ok
	1	C35H12NaO11	M+Na	C35H12O11	89.04	221.3	631.0272	0.8	1.3	29.5	even	ok
	2	C48H4N2Na	M+Na	C48H4N2	100.00	222.8	631.0267	0.3	0.5	47.5	even	ok
	3	C36H8N4NaO7	M+Na	C36H8N4O7	31.62	222.9	631.0285	2.2	3.4	34.5	even	ok
